# Quantitative description and classification of protein structures by a novel robust amino acid network: interaction selective network (ISN)

**DOI:** 10.1038/s41598-019-52766-6

**Published:** 2019-11-13

**Authors:** Shohei Konno, Takao Namiki, Koichiro Ishimori

**Affiliations:** 1 Graduate School of Chemical Sciences and Engineering, Hokkaido University, Sapporo, 060-8628 Japan; 2 Department of Mathematics, Faculty of Science, Hokkaido University, Sapporo, 060-0810 Japan; 3 Department of Chemistry, Faculty of Science, Hokkaido University, Sapporo, 060-0810 Japan

**Keywords:** Proteins, Computational biophysics, Classification and taxonomy

## Abstract

To quantitatively categorize protein structures, we developed a quantitative coarse-grained model of protein structures with a novel amino acid network, the interaction selective network (ISN), characterized by the links based on interactions in *both* the main and side chains. We found that the ISN is a novel robust network model to show the higher classification probability in the plots of average vertex degree (*k*) versus average clustering coefficient (*C*), both of which are typical network parameters for protein structures, and successfully distinguished between “all-α” and “all-β” proteins. On the other hand, one of the typical conventional networks, the α-carbon network (CAN), was found to be less robust than the ISN, and another typical network, atomic distance network (ADN), failed to distinguish between these two protein structures. Considering that the links in the CAN and ADN are defined by the interactions only between the main chain atoms and by the distance of the closest atom pair between the two amino acid residues, respectively, we can conclude that reflecting structural information from both secondary and tertiary structures in the network parameters improves the quantitative evaluation and robustness in network models, resulting in a quantitative and more robust description of three-dimensional protein structures in the ISN.

## Introduction

Proteins are biological macromolecules made up of liner chains of amino acid residues that fold into the corresponding unique three-dimensional (3D) structures comprising secondary structure elements, whereby they acquire their own functions regulated by their 3D structures. The search to understand protein structure geometries has led to the development of many experimental and theoretical methods^1–5^. To compare 3D protein structures, several sophisticated automatic secondary structure assignment programs were introduced starting more than three decades ago^6^. DSSP (Dictionary of Secondary Structure of Proteins)^7^ and STRIDE (STRuctural IDEntification)^8^ are the most widely used methods for the assignment of secondary structure from the atomic coordinates of proteins, which allow us to classify protein structures based on the secondary structure contents. Although the secondary structure contents provide us to the clearly defined criteria for protein structures, the relationship between the “whole” protein structures including tertiary structure, which can be defined as the “fold” or “topology” of proteins, and the secondary structure contents has not yet been clear. Considering that the protein functions highly depend on the “whole” structure, not on the secondary structure contents, classification based on “whole” protein structures would be more essential to discuss the functional significance of the protein structures.

Two of the most prominent protein structure classification schemes based on the “whole” protein structures, SCOP (Structural Classification Of Proteins) and CATH (Class, Architecture, Topology, Homologous superfamily)^9–13^, have been widely utilized. SCOP is the oldest structural manual classification database in which the protein structures are classified into several ‘classes’ and ‘folds’. On the first level of the hierarchy, the ‘class’ is sorted into four major classes—all-α, all-β, α + β and α/β—describing the contents of these secondary structural elements in the domain. Dominant secondary structural elements—α-helix and β-sheet—are detected by the geometry of hydrogen bonds into domains^14^. In the newer classification database, CATH, the class assignment is automatically assigned according to the ratio of the secondary structural compositions, whereas it is manually classified in the case of the protein tertiary structures^15^. However, it should be noted here that quantitative comparison of the protein secondary structures cannot be available in these approaches. This is because the ratio of the secondary structural compositions shows no clear boundaries for the specific structures as they are continuously distributed. The relationship between the ratio of the secondary structure contents and the protein classes remains unclear. Moreover, because these databases characterize the protein structures by separating several hierarchies, understanding the protein 3D structures by integrating both the secondary and tertiary structures becomes difficult.

**Figure 1:**
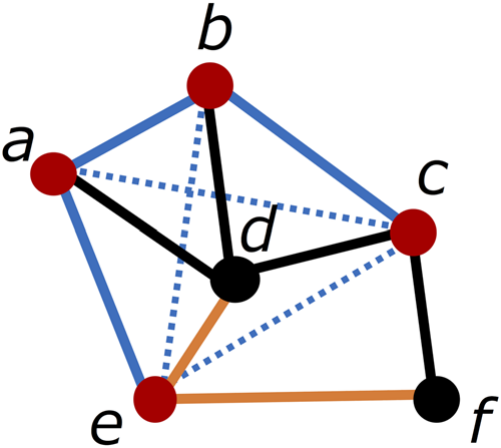
Network representation and network parameters. In an AAN, ‘vertex’ represents each amino acid residue and ‘link’ corresponds to an interaction between two specific amino acid residues. Here the network comprises six vertices—*a, b, c, d, e, f*—and links between these vertices. One of the network parameters, ‘vertex degree of one vertex *d*’, *k*_*d*_, is the number of links connected to the vertex *d*. In the figure, *d* has four links and is connected to four red vertices; *a, b, c, e* (*k*_d_ = 4). Vertex degree for each vertex (from *a* to *f*) is 3, 3, 3, 4, 3, and 2, respectively. ‘Average degree,’ *k* is the averaged vertex degree of all vertices in the protein structures (*k* = 3). ‘Clustering coefficient of *d*,’ *C*_*d*_, is the fraction of the links among the nearest neighbors of *d* (vertices established a link with *d*) to the maximum number of possible links among them. Here, *d* has four neighbors colored in red. There are three links with full blue lines between these vertices, while the maximum number of possible links between four vertices is 6 (sum of the number of links with blue continuous and dashed lines), and then ${C}_{d}=\frac{3}{6}=0.50$. ‘Average clustering coefficient’, *C* is the averaged clustering coefficient of all vertices in the protein structures. The clustering coefficient of each vertex (from *a* to *f*) is $\frac{2}{3},\frac{2}{3},\frac{1}{3},\frac{3}{6},\frac{1}{3},0$, respectively, and then $C=\frac{21}{6}/6=0.42$. ‘Distance between d and *f*’, *L*_*df*_, is the number of links on the shortest path between *d* and *f* (orange links) and then *L*_*df*_ = 2. ‘Average distance’, *L*, is the average of the distances between all vertex pairs. The total number of vertex pairs is $$(\begin{array}{c}6\\ 2\end{array})=\frac{6\times 5}{2\times 1}=15$$ and the sum of the distance between all vertex pairs is calculated as $${\sum }_{\,\begin{array}{c}i=a,\,j=b,\,i\ne j\,\end{array}}^{f}{L}_{ij}={L}_{ab}+{L}_{ac}+{L}_{bc}+\cdots =1+2+1+\cdots =21$$. Therefore, $L=\frac{21}{15}=1.4$.

Network characterization, which represents the protein 3D structure as an amino acid network (AAN), is one promising approach to providing quantitative insights into the classification of protein 3D structures. The network is a mathematical model describing complex structures as ‘vertices’ and ‘links’. Compared with previous classification approaches based on the protein secondary structure contents, AAN enables the quantitative characterization of protein geometry using the network parameters without estimating the secondary structure contents. In Fig. 1, we show a network model and calculation of the network parameters. The ‘vertices’ correspond to amino acid residues in the protein structures, while the ‘links’ represent van der Waals contacts and/or chemical interactions between two amino acid residues. We treat the AAN as a coarse-grained model of protein 3D structures characterized by vertices and links. Such AAN allows us to quantitatively argue and is *not* for analysis of protein secondary structure components, *but* for quantitative characterization of whole protein structures.

In this study, we constructed a new AAN and classified protein structures and structural domains deposited in the Protein Data Bank (PDB)^16^ by network parameters. Of the two well-known types of reported AANs, one is the Cα (α-carbon) network (CAN), in which the links are established if the distance between two Cα atoms is less than a cutoff distance, *R*_c_, which is empirically determined from 7.0 Å to 8.5 Å^5,17^. Another previously used network is the atom distance network (ADN), where the links are defined by the atom interactions between all but the hydrogen atoms in the amino acids. The ADN provides information about all van der Waals contacts between amino acid residues^5,18^. Using these AANs to characterize protein structures, previous studies have clarified several network properties of AANs^19,20^.

Many previous AAN studies have been applied to elucidate the relationship between protein domains in allosteric regulation, because the network parameters reflect the difference of global, rather than local, conformation. However, only a few studies have utilized AANs for the classification of protein structures, while an extremely limited number of studies have focused on distinguishing protein structures and classifying protein 3D structures by AAN^17,21^. Alves and Martinez, for instance, analyzed 160 low homology proteins using an AAN and classified them into four structural classes—all-α, all-β, α + β, and α/β^17^—but also showed that these four classes share similar network geometry, making it difficult to discriminate between protein structures consisting of different contents of the secondary structures such as α-helix and β-sheet structures. These previous AAN studies present two serious problems in applying network approaches to distinguish protein 3D structures.

One problem is the ambiguity in determining the optimum value of *R*_c_. Although *R*_c_ is a key factor in the development of network geometry and the characterization of network properties, in previous studies using CANs, a wide range of *R*_c_ values (mainly 7.0 Å to 8.5 Å) have been employed as the optimum value, implying ambiguity of the classification system. Previous CANs also cannot quantitatively characterize protein structures^5,17^. Links in CANs do not include the chemical properties of interactions, such as hydrogen bonds and hydrophobic or other interactions, and they are established depending only on the distance between two Cα atoms. The lack of structural information about side chains is another major problem. In CANs, the links are determined only by the distance between two Cα atoms^22^ and include no structural and chemical properties of side chains.

On the other hand, in ADNs, the optimum *R*_c_ can be uniquely determined as the distance of chemical interactions by the protein crystal structure. Although the links in ADNs are established based on the interactions involving both main chain and side chain atoms, all links are independent of the chemical properties of these interactions and thus are uniformly treated. Therefore, ADNs are also assumed to lose information about the various types of interactions. We, therefore, developed a new AAN—termed the interaction selective network (ISN)—which includes information about chemical properties of interactions, in order to quantitatively characterize and classify protein structures. The links of our ISN are determined by the distance of certain atom pairs (apart from hydrogen atoms) between two amino acid residues. We use atom pairs involved in hydrogen bonds, hydrophobic interactions, disulfide bonds, ionic interactions and covalent bonds. The ISN as a model of protein structures has the advantage that interactions involved in *both* main chain and side chain atoms are reflected. The ISN has thus enabled us to discriminate between all-α and all-β proteins by their geometry, not based on the ratio of the secondary structure elements. We also confirmed the difficulty of characterizing the protein secondary structures with previously used CANs and ADNs. Although we found that a CAN using *R*_c_ = 5.5 Å appears to distinguish between all-α and all-β proteins, a small deviation of *R*_c_ from 5.5 Å resulted in a less clear discrimination with a flip in the clusters of the protein structures, implying that the CAN is a less robust network than the ISN in terms of *R*_c_. The ISN is, therefore, a more quantitative and robust AAN, which we expect will be widely used for quantitatively characterizing and classifying protein 3D geometries.

## Results

### Distinguishing protein structures by the interaction selective network (ISN)

As previously reported^5,17^, the network models have several common parameters^13^ as listed in Table 1. To find out the correlation between these parameters to discriminate protein structures, we tentatively fix the *R*_c_ values for the interactions between the amino acid residues to determine the “links”. The ISN comprises five types of interactions—hydrogen bonds, hydrophobic interactions, disulfide bonds, ionic interactions and covalent bonds. For hydrogen bonds, the initial *R*_c_ value was 3.5 Å, corresponding to the maximum donor-acceptor distance, as previously used^23,24^. In the case of hydrophobic interactions, *R*_c_ was 5.0 Å between the side chain carbon atoms in the following hydrophobic residues: Ala, Val, Leu, Ile, Met, Phe, Trp, Pro and Tyr^25^. The initial *R*_c_ value of a disulfide bond was 2.2 Å between the sulfur atoms, while that of the ionic bond was 6.0 Å between the side chain nitrogen and oxygen atoms in the following ionic residues: Arg, Lys, His, Asp and Glu. The covalent bond is established if there are two consecutive amino acid residues on the amino acid sequence. Although we calculated correlations between all network parameters as listed in Table 1, a significant correlation to discriminate protein structures was only observed between average vertex degree (*k*) and average clustering coefficient (*C*) (correlations between other parameters are shown in Fig. S1).

**Table 1:** Network parameters in this study.

Network parameters	Symbol	Description
Number of vertices	*N*_Vertices_	The number of vertices in the network.
Number of links	*N*_Links_	The number of links in the network.
Average vertex degree	*k*	Average degree for all vertices. Degree of a vertex is the number of links connected to the vertex.
Average clustering coefficient	*C*	Average of clustering coefficient for all vertices. Clustering coefficient of a vertex is the fraction of links that exist among the nearest neighbours of each residue to the maximum number of possible links among them.
Average distance	*L*	Average of network distance for all vertex pairs. Network distance is the number of links on the shortest path between vertices.
Vertex assortativity	—	The correlation of the degree between vertices adjacent to each other.
Maximum vertex degree	*k*_max_	The maximum degree in the network.

To construct the ISN, the optimum *R*_c_ values for these interactions except for the covalent bonds should be determined. Although determining the optimum set of the *R*_c_ values for these four interactions would not be simple and require complicated procedures, it should be noted here that, among five types of interactions, hydrogen bonds and hydrophobic interactions are primary interactions in protein structures and, most of the links in the ISN are, therefore, established by hydrogen bonds and hydrophobic interactions. Such primary contributions of hydrogen bonds and hydrophobic interactions to the links in the ISN suggest that the *R*_c_ values for hydrogen bonds and hydrophobic interactions are essential to discriminate protein structures by the ISN. We, therefore, constructed the ISN with wide range of *R*_c_ of hydrogen bonds and hydrophobic interactions to determine the optimum *R*_c_ values, and then recalculated their network parameters (Table 1) to discriminate protein structures.

**Figure 2:**
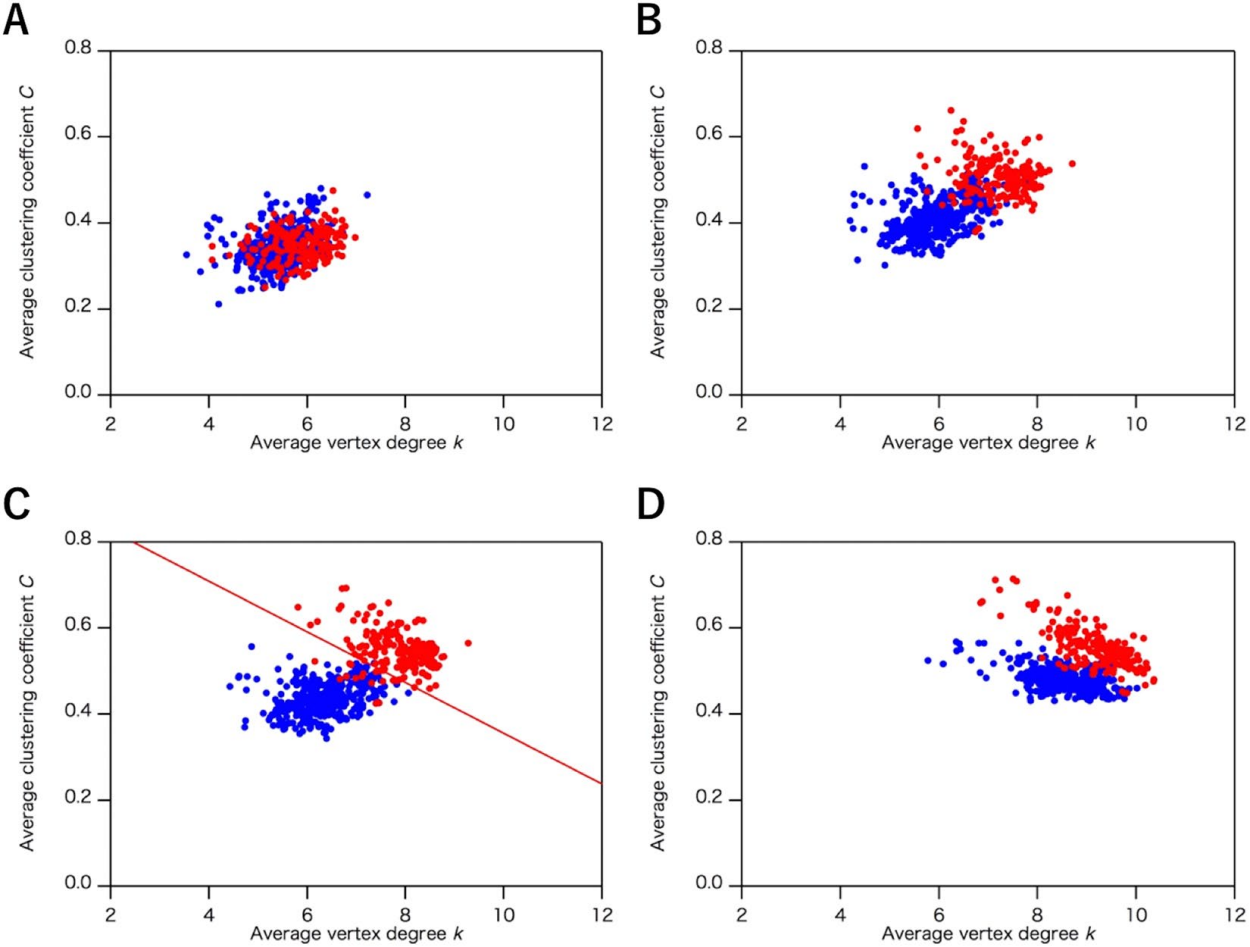
The plots of the average clustering coefficient (*C*) and the average vertex degree (*k*) in the interaction selective network (ISN) with different cutoff value (*R*_c_) of the hydrogen bonds. The distribution was calculated by using the common data set of the three network models (1,520 structures). The *R*_c_ value is set as (**A**) 3.2 Å, (**B**) 3.4 Å, (**C**) 3.8 Å, and (**D**) 5.0 Å. Plots of all-α and all-β proteins are colored in red and blue, respectively. In (**C**), discriminant line, $C=0.915-0.0564\,k$ was determined by the logistic regression analysis. The ratio of correctly distinguished structures is 0.9323 and 0.9843 for all-α and all-β proteins, respectively.

In Fig. 2, we show the effects of *R*_c_ for hydrogen bonds on the classification of the all-α and all-β proteins (the number of structures and list of PDB entries are shown in Table 2 and S1, respectively). We constructed ISNs with *R*_c_ ranging from 2.0 to 5.0 Å for hydrogen bonds and compared the distributions of *k* and *C* under the conditions that fix *R*_c_ for other interactions. Under *R*_c_ = 3.2 Å, the distributions of *k* and *C* for the all-α and all-β proteins overlapped (Fig. 2A). Ranging from *R*_c_ = 3.4 Å to *R*_c_ = 3.8 Å, the distributions were segregated with increasing *R*_c_ (Fig. 2B,C), while with *R*_c_ > 3.8 Å the distributions were less segregated (Fig. 2D). In the region from *R*_c_ = 3.4 Å (Fig. 2B) to *R*_c_ = 3.8 Å (Fig. 2C), *k* and *C* of the all-α proteins were increased, whereas those of the all-β proteins were almost unchanged (Fig. 2B,C), reflecting the different geometry between α-helix and β-sheet structures (*k–C* plots with *R*_c_ ranging from 2.0 to 5.0 Å with 0.2 Å intervals are shown in Fig. S2 in the Supplementary section).

**Table 2:** The number of protein structures used for the Cα network (CAN), the atom distance network (ADN), and the interaction selective network (ISN).

	CAN	ADN	ISN	Common
All-α	247	254	207	199
All-β	557	557	454	448
α + β	647	664	551	543
α/β	399	415	344	330
Total	1,850	1,890	1,556	1,520

The common data set in the three network models were used for the further analysis and the testing data set to estimate the classification probability.

**Figure 3:**
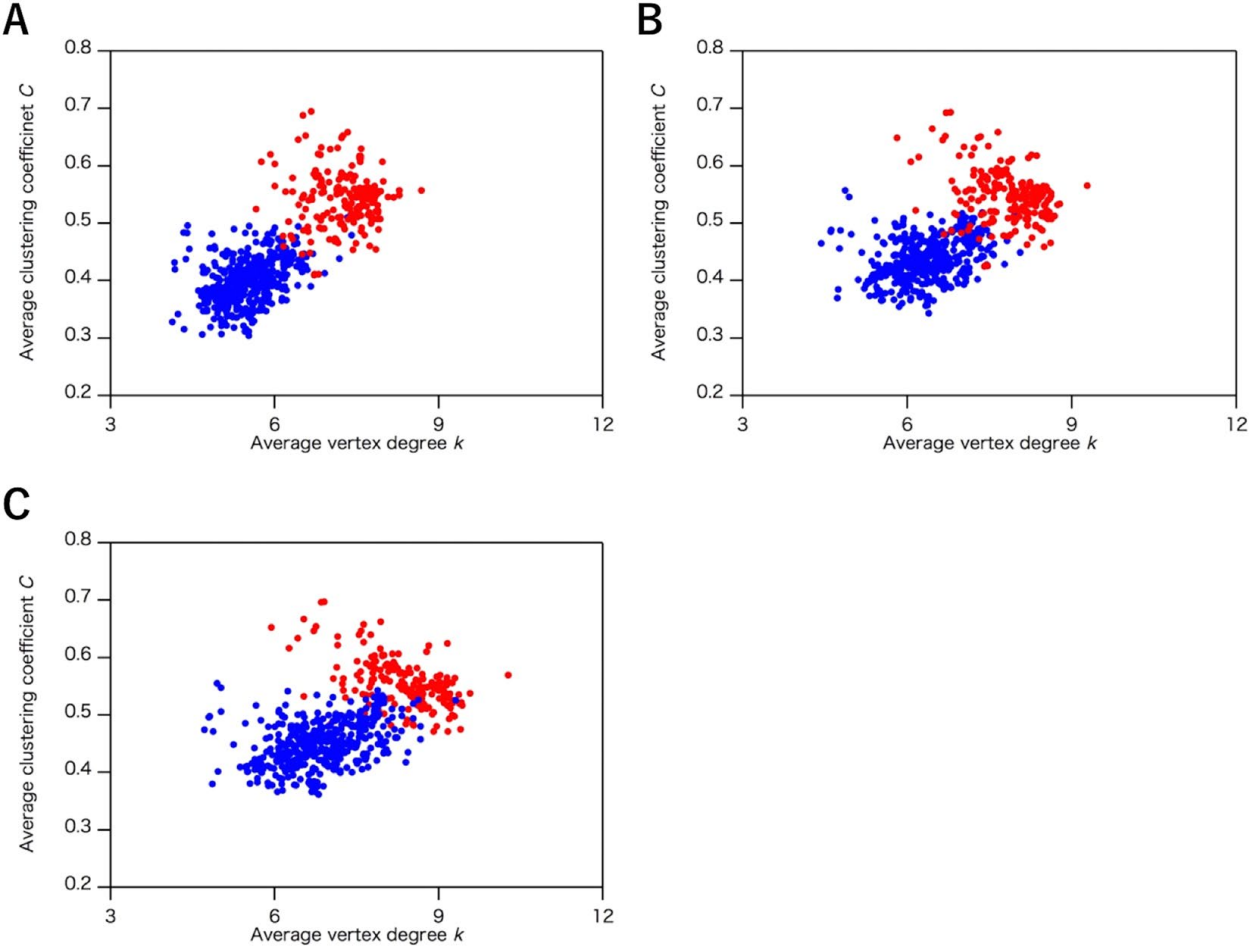
The plots of the average clustering coefficient (*C*) and the average vertex degree (*k*) in the interaction selective network (ISN) with different cutoff value (*R*_c_) of the hydrophobic interactions. The distribution was calculated by using the common data set of the three network models (1,520 structures). The *R*_c_ value is set as (**A**) 4.0 Å, (**B**) 5.0 Å, and (**C**) 6.0 Å. Plots of all-α and all-β proteins are colored in red and blue, respectively.

We also examined the effect of *R*_c_ for hydrophobic interactions, another primary interaction in protein structures, on the classification of the all-α and all-β proteins (the number of structures is shown in Table 2). Figure 3 displays the *k*–*C* plots of the all-α and all-β proteins with *R*_c_ from 4.0 to 6.0 Å (*k–C* plots with *R*_c_ ranging from 3.5 to 6.5 Å with 0.2 Å intervals are shown in Fig. S3 in the Supplementary section). In contrast to the case for the hydrogen bonds, the network parameters remained almost unperturbed by changing *R*_c_ of hydrophobic interactions around 5.0 Å (Fig. 3B). Such low sensitivity of the network parameters to *R*_c_ indicates that the ISN geometry is almost independent of *R*_c_ for hydrophobic interactions, which in turn suggests that *R*_c_ of hydrogen bonds is the dominant factor for the network geometries of protein structures. For other interactions, only a few links are included in protein structures, and the contribution of their *R*_c_ values to the network structures and parameters would be quite limited. The *R*_c_ values for disulfide bonds, and ionic interactions were, therefore, fixed at 2.2 Å, and 6.0 Å, respectively, for the further analysis.

### Characterizing protein structural classes by the interaction selective network (ISN)

**Figure 4:**
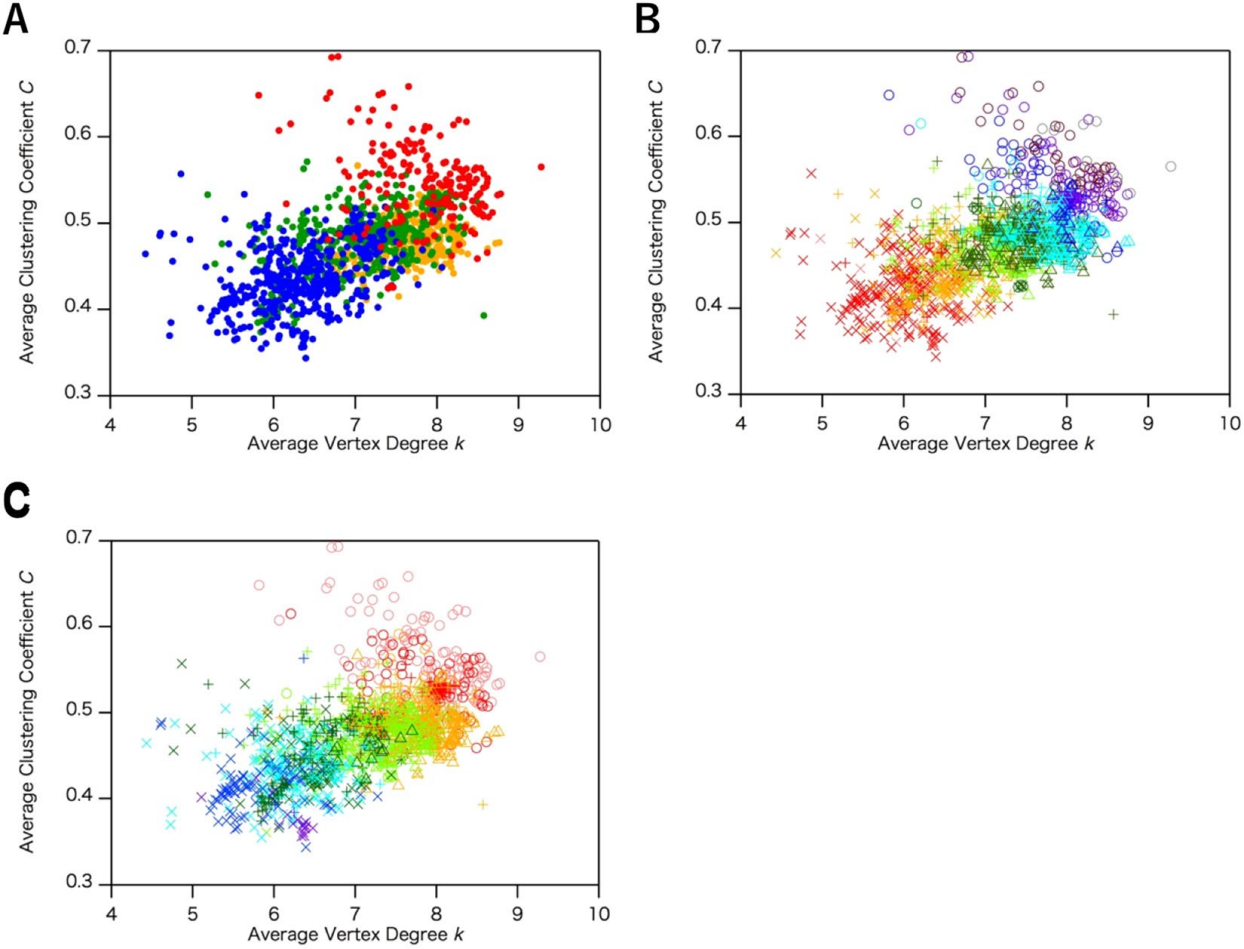
(**A**) The plot of average clustering coefficient (*C*) and average vertex degree (*k*) in the interaction selective network (ISN). The distribution was calculated by using the common data set of the three network models (1,520 structures). Protein structures are categorized as all-α (red), all-β (blue), α + β (green), and α/β (orange); (**B**,**C**) The *k*–*C* plots identical to (**A**) with visualizing (**B**) α-helix and (**C**) β-sheet contents. Secondary structure contents are shown with colored plots as follows; 0% (pink), 1–10% (red), 11–20% (orange), 21–30% (yellow green), 31–40% (green), 41–50% (light blue), 51–60% (blue), 61–70% (purple), 71–80% (deep purple), 81–90% (grey), and 91–99% (black). In (**B**) and (**C**), these protein structures are classified as all-α (◦), all-β (×), α + β (+), and α/β (∆).

In the previous section, we focused on the all-α and all-β protein classes, without examining the classification of the additional two classes, α + β and α/β. In Fig. 4A, we illustrate the protein structures categorized into these four classes as examined by the ISN, where the distribution of *k* and *C* is shown. The distributions of all-α (red) and all-β proteins (blue) are well-separated under the conditions, *R*_c_ (hydrogen bonds) = 3.8 Å, and *R*_c_ (hydrophobic interactions) = 5.0 Å. As we expected, the distribution of the α + β and α/β proteins are overlapped with the boundary region between the all-α and all-β proteins, because α + β and α/β classes have both α-helix and β-sheet components. It should be noted here that some proteins classified into the all-α or all-β classes are located in the boundary region between the distributions of the all-α and all-β proteins in the *k–C* plots, as observed for the α + β and α/β proteins. The X-ray structural analysis reported that some inconsistent proteins have both α-helix and β-sheet structures^26^. For instance, type III antifreeze protein RD1 from an Antarctic eelpout (PDB ID: 1ucs) is classified as an all-β protein in SCOP, whereas *k* and *C* (*k* = 8.19, *C* = 0.506) for this protein in the ISN can be plotted in the region for α/β proteins, not all-β proteins (Fig. 4A). Based on the X-ray crystal structure of this protein^26^, the α-helix and β-sheet contents are 20% and 25%, respectively, and comparable contents of the α-helix structure support the structural assignments by our analysis.

We also found that the ratio of the secondary structure content correlates with the distribution of the *k*–*C* plot. In Fig. 4B,C, we show the dependence of *k* and *C* on the secondary structure contents. Larger *k* and *C* values of the ISN were calculated by increasing the α-helix contents of proteins, whereas the corresponding decrease in the β-sheet contents were less clear. These results suggest that the ISN detects the geometry of the α-helix components and reflects the ratio of α-helix in the network parameters, *k* and *C*, allowing us to discriminate between the all-α and all-β proteins.

As illustrated in Fig. 4A, some of the protein classes are overlapped in the *k*–*C* plot of the ISN. The α/β proteins (mainly from 7.2 to 8.3 for *k* and from 0.44 to 0.52 for *C*) had relatively higher *k* values, as observed for the all-α proteins (mainly from 7.4 to 8.6 for *k* and from 0.48 to 0.59 for *C*), and medial *C* values, which were observed for the all-α proteins and the all-β proteins (mainly from 5.8 to 7.4 for *k* and from 0.37 to 0.50 for *C*). On the other hand, the α + β proteins (mainly from 6.3 to 8.3 for *k* and from 0.43 to 0.53 for *C*) showed a wide range of distribution of *k* and *C*, which overlapped with those for the all-α (overlapped range: from 7.4 to 8.3 for *k* and from 0.48 to 0.53 for *C*) as well as those for the all-β proteins (overlapped range: from 6.3 to 7.4 for *k* and from 0.43 to 0.50 for *C*). Although the different distributions of the *k*–*C* plot between the α + β and the α/β proteins were roughly proportional to the α-helix content (Fig. 4B), these overlaps on the *k*–*C* plots suggest that the geometry of the secondary structure elements should also be considered for proteins having both α-helix and β-sheet structures.

### Comparison of the interaction selective network (ISN) with previously used amino acid networks (AANs)

**Figure 5:**
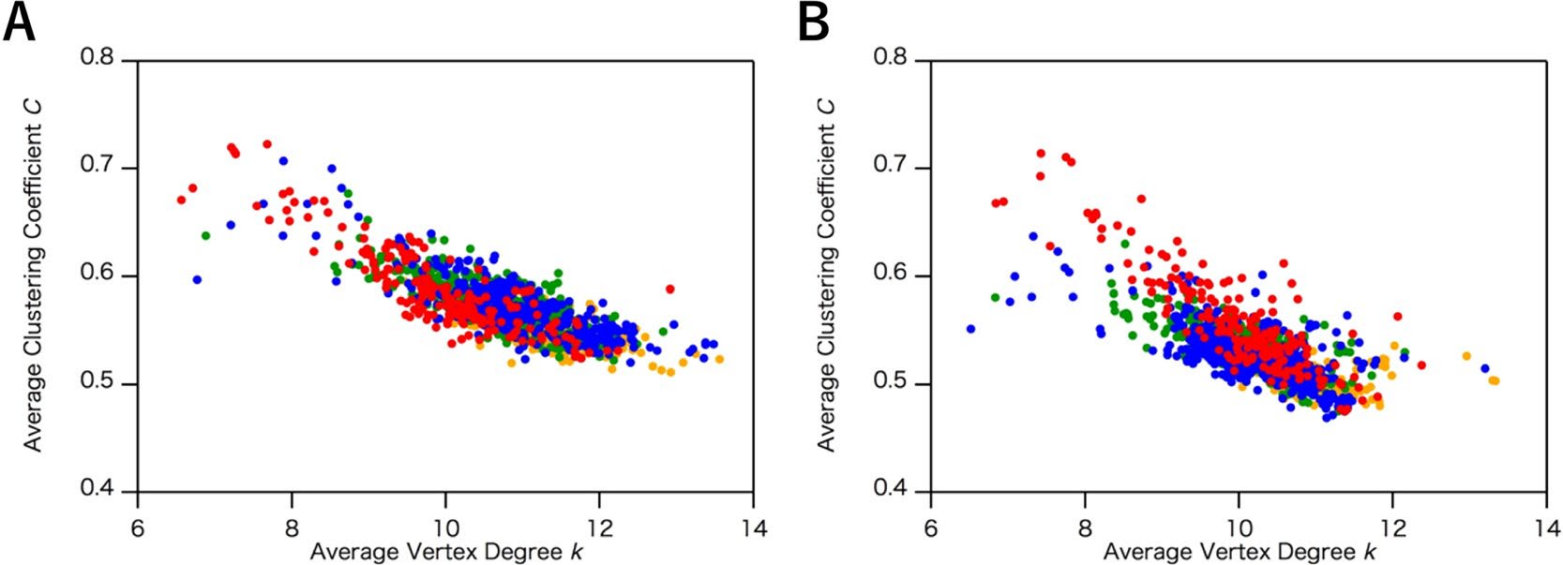
The plot of average clustering coefficient (*C*) and average vertex degree (*k*) in (**A**) CAN (*R*_c_ = 8.5 Å) and (**B**) ADN. The distribution was calculated by using the common data set of the three network models (1,520 structures). The protein class of each protein is shown with colored plots as follows: all-α (red), all-β (blue), α + β (green), and α/β (orange).

To confirm the validity of the ISN for characterizing protein structures, we compared the network properties of the ISN with those of the CAN and the ADN. For protein structures, *k* and *C* were calculated and classified according to their protein classes. In contrast to the ISN, the CAN (*R*_c_ = 8.5 Å) and the ADN (*R*_c_ = 5.0 Å) share a similar distribution of the network parameters in protein structures, regardless of the different classes as show in Fig. 5A,B, respectively. This difference in distribution arises from the definition of the links in the network models. In the CAN, the links are defined by the distance between two α-carbons of the main chain without interactions between the side chains. Although the ADN includes interactions involving both main and side chain atoms, the distribution of the *k*–*C* plot is still significantly different from that of the ISN. The treatment of van der Waals interactions comprises the largest difference between these two models. In the ADN, all van der Waals interactions are counted as links, but the links in the ISN are defined only by van der Waals interaction between hydrophobic residues. The ISN, therefore, detects only critical interactions as links, without recognizing weakly interacting contacts with the neighbor residues in their 3D structures. This property enables us to discriminate the difference in geometry between protein structures.

**Figure 6:**
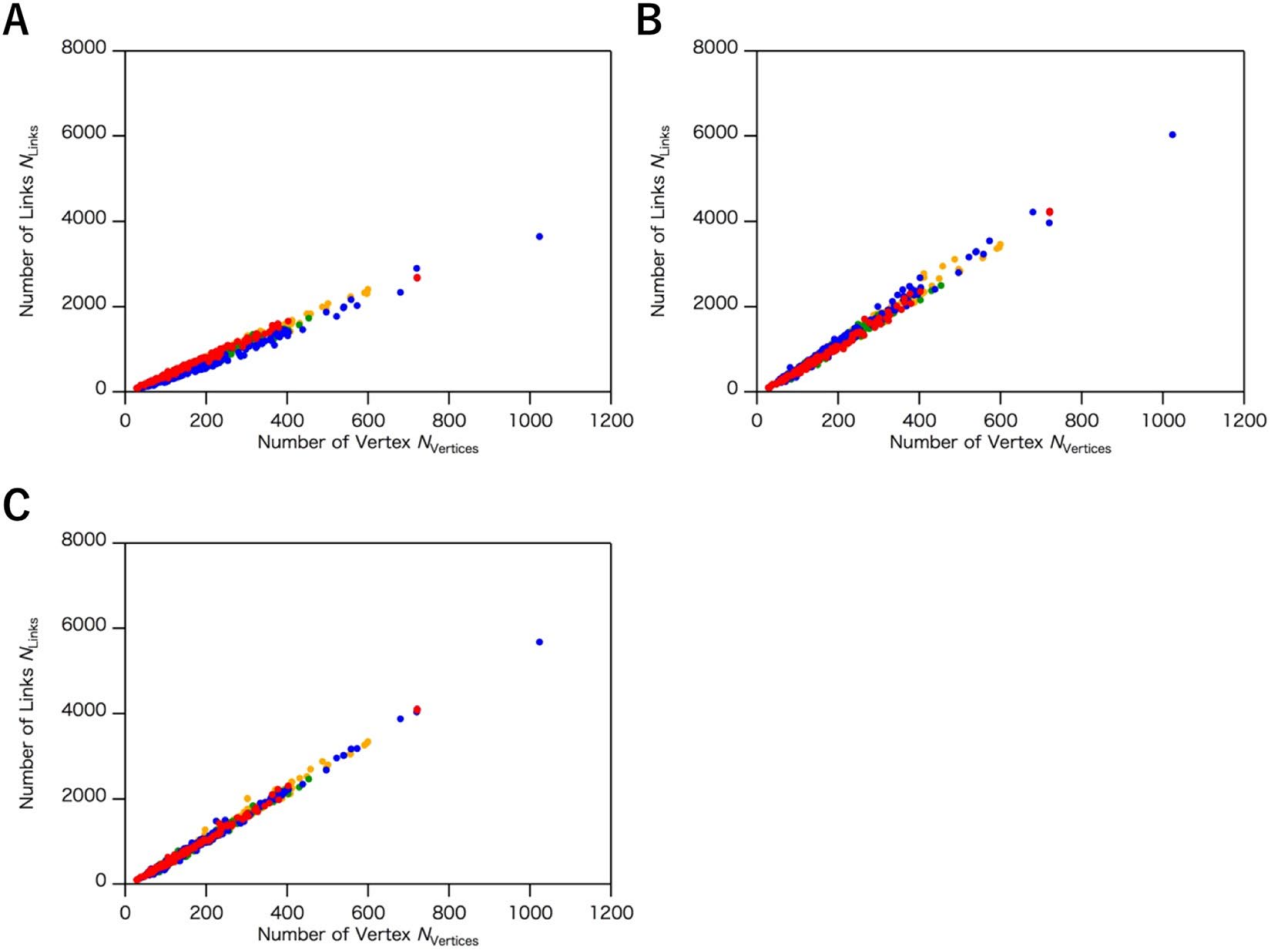
The plot of the number of links (*N*_Links_) versus the number of vertices (*N*_Vertices_) in (**A**) ISN, (**B**) CAN (*R*_c_ = 8.5 Å), and (**C**) ADN (*R*_c_ = 5.0 Å). The distribution was calculated by using the common data sets. The class of each protein is shown with colored plots as follows: all-α (red), all-β (blue), α + β (green), and α/β (orange).

Other notable network parameters are the numbers of vertices, *N*_Vertices_, and links, *N*_Links_. As clearly shown in Fig. 6, we detected a higher correlation between *N*_Vertices_ and *N*_Links_. The slopes of *N*_Links_ – *N*_Vertices_ plots are summarized in Table 3. In the ISN, the linear relationships with clear differences in the slopes of the four protein classes—all-α, α/β, α + β and all-β protein structures, in descending order of the slopes, were observed. Steeper slopes correspond to the protein structures that have more links per vertex than the other structures. An increased *N*_Links_ implies a more crowded network, corresponding to larger *k* and *C* values, consistent with the fact that the hydrogen bond network of the α-helix is a spatial structure with many interacting atoms, whereas the β-sheet is a more planar structure with a lower number of interacting atoms. On the other hand, in the case of the CAN and the ADN, it is difficult to detect the correlations between the slopes and the protein classes, as shown in Fig. 6B,C, respectively.

**Table 3:** The slopes of the number of links(*N*_Links_) – the number of vertices (*N*_Vertices_) plots for all-α, all-β, α + β and α/β proteins in the Cα network (CAN), the atom distance network (ADN), and the interaction selective network (ISN) in the individual whole data set. The standard deviation is used for error in each slop.

	CAN	ADN	ISN
All-α	5.46 ± 0.03	5.38 ± 0.02	3.96 ± 0.02
All-β	5.77 ± 0.02	5.38 ± 0.01	3.41 ± 0.01
α + β	5.51 ± 0.01	5.25 ± 0.01	3.66 ± 0.01
α/β	5.78 ± 0.01	5.49 ± 0.01	3.88 ± 0.01

### Reexamination of discrimination between all-α and all-β protein by Cα network (CAN)

**Figure 7:**
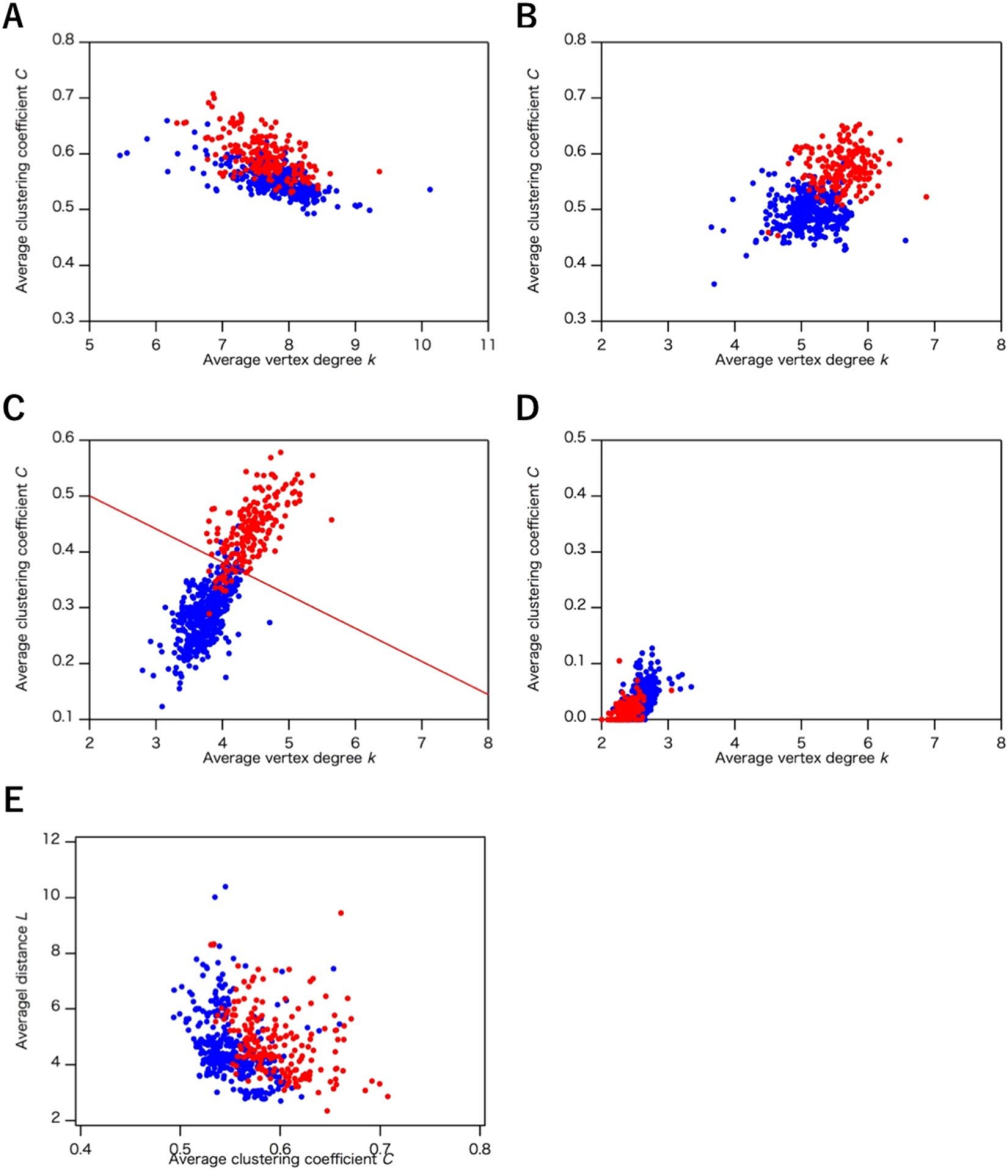
Average clustering coefficient (*C*)–average vertex degree (*k*) plots (**A**–**D**) and average distance *L*–*C* plots (**E**) in CAN. The distribution was calculated by using the common data set of the three network models (1,520 structures). The cutoff value (*R*_c_) of CAN is (**A**), (**E**) 7.0 Å, (**B**) 6.0 Å, (**C**) 5.5 Å, and (**D**) 5.0 Å. All-α protein structures are represented as red circles and all-β protein structures are shown as blue circles. In (**C**), discriminant line, $C=0.619-0.0593\,k$ was determined by the logistic regression analysis.

Previous sections demonstrated that we successfully classified the protein structure using the ISN, but Bagler and Sinha also reported that they could distinguish all-α and all-β protein structures using the CAN (*R*_c_ = 7.0 Å)^20^. Their results display a clear separation of all-α and all-β proteins in the *L*–*C* plots, in contrast to our result (*R*_c_ = 8.5 Å, Fig. 5A). To confirm the effect of *R*_c_ on the distribution of network parameters *k*, *C* and *L* in the CAN, we reconstructed the CAN (*R*_c_ = 5.0 Å ~ 8.5 Å) and we show the *k*–*C* and *L*–*C* plots in Fig. 7. In this range of the *R*_c_ values, disconnected components were not obtained because these *R*_c_ values were larger than the distance between the corresponding adjacent α-carbon atoms. The distributions of *k* and *C* indicate that all-α and all-β protein structures were not discriminated at *R*_c_ = 7.0 Å (Fig. 7A), while lowering *R*_c_ to under 6.0 Å enabled us to separate the two classes (Fig. 7B). The two classes appear to be separated at *R*_c_ = 5.5 Å (Fig. 7C). However, when lowering *R*_c_ to under 5.5 Å, *C* dramatically decreased with decreasing *R*_c_, showing a flip in the clusters of the all α- and all β-proteins and resulting in the loss of clustering structures (Fig. 7D). Such a drastic change in the distribution of the *k*–*C* plots by the small perturbation of *R*_c_ indicates the instability of the CAN as a network model, corresponding to a less robust network than the ISN.

Previous study^20^ reported that the clusters of all-α and all-β proteins were separated in the *L–C* plots of the CAN (*R*_c_ = 7.0 Å), but the *L*–*C* plots of the CAN (*R*_c_ = 7.0 Å) we calculated here indicate that the distribution of all-α and all-β proteins are overlapped as shown in Fig. 7E, which is in contrast to the previous study. This discrepancy between the two results is due to the small number of the data sets in previous study. The previous study used only 20 protein structures for each protein class^20^, whereas we used 247 all-α and 557 all-β protein structures in our study (Table 2), showing that our data set is sufficiently large to display the overlapping of plots of between all-α and all-β proteins and the number of the data sets is essential. From the above discussion, the CAN would not be a robust network model to characterize and classify protein structures.

## Discussion

As shown in the results, significant differences between all-α and all-β proteins were detected in average vertex degree, *k*, and average cluster coefficient, *C*, in the ISN and the CAN. To confirm the validity of the classification based on these network models, the logistic regression^27^, which is one of widely used statistical methods to examine the validity of the classification and discrimination, was applied to obtain an optimum classification line which does not so much affected by outlier values. Using the optimum classification line, we calculated classification probability given by the number of exactly classified into all-α (all-β) proteins over the number of all-α (all-β) proteins. In this analysis, the common data set for the three network models (Table 2) and the selected data set (Table 4) were utilized as the testing data set and the learning data set, respectively. The data of the selected data set were extracted from the common data set by applying the additional screening (see “Data sets of protein structures” in the Methods section.). Before applying the logistic regression, differences among the distributions for each AAN are estimated by statistical test. Using Kolmogorov-Smirnov test on the learning dataset for the ISN (*R*_c_ = 3.8 Å), the CAN (*R*_c_ = 8.5 Å) and the ADN, null hypothesis that the two distributions of the average degree (*k*) in both all-α proteins and all-β proteins are identical was tested with a significance level of 0.05. The hypothesis was rejected for the ISN (*R*_c_ = 3.8 Å) with *p-*value < 2.2 × 10^−16^, also rejected for the CAN (*R*_c_ = 8.5 Å) with *p-*value = 0.01588, but not rejected for the ADN with *p*-value = 0.1445. Null hypothesis that the two distributions of the mean clustering coefficient (*C*) are identical was also tested. The hypothesis was rejected for the ISN (*R*_c_ = 3.8 Å) with *p-*value < 2.2 × 10^−16^, but not rejected for the CAN (*R*_c_ = 8.5 Å) with *p-*value = 0.331, and rejected for the ADN with *p-*value = 3.7 × 10^−7^. By the results of statistical test, all-α protein was distinguished from all-β protein by significantly smaller *p*-values for each distribution of *k* and *C* only in the ISN (*R*_c_ = 3.8 Å). On the other hand, the null hypothesis was rejected with *p-*value < 2.2 × 10^−16^ for both *k* and *C* for the CAN (*R*_c_ = 5.5 Å) as observed for the ISN (*R*_c_ = 3.8 Å). These results show that both the ISN and the CAN (*R*_c_ = 5.5 Å) on the learning dataset are significantly different from previously studied AAN, the ADN and the CAN (*R*_c_ = 8.5 Å), and all-α proteins and all-β proteins are classified by the logistic regression on the ISN (*R*_c_ = 3.8 Å) and the CAN (*R*_c_ = 5.5 Å). The optimum classification line for the ISN (*R*_c_ = 3.8 Å) in Fig. 2C was given by $C=0.915-0.0564\,k$ on the *k–C* plane, and, for the CAN (*R*_c_ = 5.5 Å) in Fig. 7C, $C=0.6191-0.0593\,k$ was obtained. For the ISN (*R*_c_ = 3.8 Å), the classification probabilities on all-α and all-β proteins are 0.9323 and 0.9843, respectively. On the other hand, those on all-α and all-β proteins in the CAN (*R*_c_ = 5.5 Å) were 0.8906 and 0.9641, respectively. For both the ISN (*R*_c_ = 3.8 Å), and the CAN (*R*_c_ = 5.5 Å), the classification probabilities were over 0.88, implying that the classifications of all-α and all-β proteins in the *k–C* plots of the ISN and the CAN are statistically significant.

**Table 4:** The number of protein structures used for the Cα network (CAN), the atom distance network (ADN), and the interaction selective network (ISN) in the learning data set to estimate the classification probability.

	CAN	ADN	ISN
All-α	59	60	52
All-β	83	84	71
α + β	155	157	131
α/β	161	163	135
Total	458	464	389

As clearly shown in the Results section, although both the ISN and the CAN (*R*_c_ = 5.5 Å) were able to distinguish between all-α and all-β protein structures, the ISN and the CAN differ in terms of three points, as follows: the robustness, the average degree for all vertices, *k*, and the distribution of the network parameters. Robustness in network models is defined by the capability of maintaining similar network geometry with a wide range of parameters. We, therefore, use ‘robustness’ as the capability of distinguishing all-α and all-β protein structures within a wide range of *R*_c_ values, corresponding to a stable model, while less robust networks are unstable models drastically perturbed by small deviations of the network parameters. A notable difference in the robustness between the CAN and the ISN resides in the comparison between the distributions of the *k*–*C* plots. The all-α and all-β proteins were distinguished with a wide range of *R*_c_ from 3.4 Å to 5.0 Å in the ISN, while the CAN was able to distinguish between all-α and all-β protein structures only around *R*_c_ = 5.5 Å. The CAN lost clear discrimination when *R*_c_ was lower than 5.0 Å and flipping was observed for the clusters of the two protein structures, where *C* became approximately 0 and *k* was between 2 and 3 (Fig. 7D), showing that the CAN is less robust than the ISN in terms of *R*_c_.

A comparison of the *k* values between the CAN and the ISN also indicated a significant difference between the two amino acid networks. The *k* value of protein structures in the CAN (*R*_c_ = 5.5 Å) ranged from 3 to 5 (Fig. 7C), thus exhibiting three to five links per vertex. This number of links per vertex corresponds to the number of interactions around Cα atoms. Among these links, two were derived from the links to the neighbor residues by covalent bonds. For the remaining one to three links, most would be hydrogen bonds involved in the main chain atoms. If a hydrogen bond is established between two residues by their main chain atoms, their Cα atoms are also close to each other. These hydrogen bonds reflect the protein secondary structures and, therefore, most of the links in the CAN (*R*_c_ = 5.5 Å) include interactions only in main chain atoms. In the case of the ISN, five to nine links were exhibited per vertex (Fig. 4A), implying an additional three to seven interactions besides the two covalent bonds. These additional three to seven links cannot be accounted for only by interactions involved in main chain atoms, indicating that some of the links are derived from interactions involved in side chain atoms. The links in the ISN, therefore, comprise interactions involved in *both* main and side chain atoms and the ISN includes structural information about the secondary and tertiary structures, corresponding to the interactions mediated by main and side chain atoms, respectively. This is in contrast to the CAN (*R*_c_ = 5.5 Å), wherein links only reflect the secondary structures from interactions in the main chain atoms.

As discussed above, the CAN (*R*_c_ = 5.5 Å) is based on the information about protein secondary structures reflecting hydrogen bonds between main chain atoms. On the other hand, the ISN includes additional information about interactions involved in the side chain atoms. Since the side chain atoms often mediate van der Waals contacts and hydrophobic interactions between two residues located on separated positions on the primary sequence, thus reflecting the tertiary structures of proteins, the links in the ISN include the structural information from the tertiary structures of proteins. The scattered distribution in the *k*–*C* plots in the ISN, compared to that in the CAN, also suggests that, not only the structural information from the secondary elements, but also additional structural information from the tertiary structures are reflected in the distribution. The CAN (*R*_c_ = 5.5 Å), therefore, describes the protein structures only according to the secondary structures, whereas the ISN categorized protein structures based on both their secondary and their tertiary structures.

To get further insights into the mathematical basis for the discrimination of protein structures in the two network models, we performed one of the typical multivariate analyses, principal component analysis, of these network models. To apply the method to the *k–C* plots of the ISN and the CAN, we retrieved eigenvalues and eigenvectors of correlation matrix from target multivariate data and, then, the direction of eigenvector of the maximal eigenvalue was selected as the first principal component. In the *k–C* plots, the data are two-dimensional and the correlation matrix is two-by-two. Based on the correlation matrix, the two orthonormal directions, the direction of the eigenvector of maximal eigenvalue and the direction of the second eigenvector were determined. Variance along each direction shows the contribution of the direction to the correlation of two variables, *k* and *C*. The first direction is referred to as the principal component one (PC1) and the second direction as the principal component two (PC2). In the CAN (*R*_c_ = 5.5 Å), the standard deviations of PC1 and PC2 are calculated to 0.1662 and 0.03614, respectively. The proportion of the variance in PC1 and PC2 is, therefore, 0.9549 and 0.04512, respectively, indicating that most of the information (more than 95%) to discriminate the protein structures on the *k–C* plots in the CAN is primarily based on PC1. Thus, the discrimination of protein structures in the CAN depends on only one parameter of the protein structures, corresponding to the secondary structures. On the other hand, the standard deviations of PC1 and PC2 in the ISN are 0.1181 and 0.06049, respectively, and the proportion of variance in PC2 was enhanced to 0.20773, while that in PC1 was decreased to 0.7923. In the ISN, over 20% of the information is, therefore, included along PC2, and the second principal component significantly contributed to the discrimination of the *k–C* plots in the ISN, supporting the contribution of the information of the tertiary structure to the correlation between *k* and *C* in the ISN. Considering the stability of the model depends on the number of the principal components, the significant contribution of the second principal component to the discrimination of protein structures on the *k–C* plots also suggests that the ISN is a more stable and robust network model than the CAN.

We thus successfully categorized protein structures using the stable and robust model, the ISN. Two network parameters, *k* and *C*, enabled us to distinguish between all-α and all-β protein structures. By exploring the optimum *R*_c_ for the discrimination of protein structures, we determined that the ISN is more robust than the CAN, while the wider range of the distribution of *k* and *C* and the multivariate analysis in the ISN suggests that the ISN has additional structural information from protein tertiary structures. On the other hand, as clearly shown in Figs 5B and 6C, another network model, ADN, cannot distinguish between all-α and all-β proteins in the *k–C* and *N*_link_*–N*_vertices_ plots, regardless of the *R*_c_ value. The distributions of other network parameters including *L*, *k*_max_, and vertex assortativity also indicated no clear differences between all-α and all-β protein structures (Fig. S1). Therefore, the ISN provides a more quantitative and robust description of protein 3D structures, by reflecting both secondary and tertiary protein structures.

As discussed above, the ISN, based on whole protein structures including the secondary and tertiary structures, would be a promising coarse-grained model for quantitatively categorizing protein structure without estimating the secondary structure contents, which will provide new insights into the classification of the protein. By characterizing the protein structures by average vertex degree, *k*, and average clustering coefficient, *C*, the ISN can classify the protein structures into the group defined by the specific *k* and *C* values. Considering that the whole protein structure is one of the major determinants of the protein functions, the classification based on the tertiary structure allows us to find new biological functions of the proteins showing similar secondary structure contents but different tertiary structures. Furthermore, the classification of the protein structures by the network model and characterization of protein structures by the network parameters also contribute to the mathematical understanding of protein structures, which will pave the way for designing artificial proteins with new structures never found in nature.

## Methods

### Construction of amino acid network (AAN)

The AANs we examined here use the structural data of native-state protein, structural domains, and some partial protein structures, as available in PDB^16^, and each amino acid residue of the protein molecule is defined to be taken as a “vertex” in AANs. If we identify that two amino acid residues contact each other in a protein structure, we assume that a “link” is established. All interactions between the residues are identified as links. We used three types of AANs with differing definition of the links. In the CAN, two residues are connected (a link between two vertices is established), if the distance between their Cα atoms is less than *R*_c_. The links of the ADN are determined by the distance of the closest atom pairs between two amino acid residues. Because data sets of the protein structures determined by X-ray crystallography do not contain the atomic coordinates of hydrogen atoms, hydrogen atoms are ignored. If the distance is less than *R*_c_ = 5.0 Å, these two amino acid vertices are connected; otherwise, they are disconnected. *R*_c_ is defined as the distance of van der Waals contacts^28,29^ in the ADN. In our novel introduction of the ISN, the links are determined by the distance as defined in the ADN. However, to establish the links, we only use atom pairs involved in hydrogen bonds, hydrophobic interactions, disulfide bonds, ionic interactions and covalent bonds. Hydrogen atoms, as in the ADN, are ignored. *R*_c_ in the ISN is determined based on the cutoff value used in Protein Interaction Calculator (PIC)^30^. PIC is a server computing various interactions in a protein structure based upon the coordinate set of the 3D structure of the protein.

These AANs are characterized by following several parameters^14^, as shown in Table 1. The network parameters widely used for previous AANs are average vertex degree *k*, average clustering coefficient *C*, and average path length *L*, as shown in Fig. 1 ^5,17,31–33^. The *k* value of a protein structure is defined as

$$\begin{array}{c}{\boldsymbol{k}}=\frac{{\bf{1}}}{{\boldsymbol{N}}}\mathop{\sum }\limits_{{\boldsymbol{i}}={\bf{1}}}^{{\boldsymbol{N}}}{{\boldsymbol{k}}}_{{\boldsymbol{i}}}\end{array}$$

where *N* is the number of amino acid residues, and the vertex degree, *k*_*i*_, is the connected number of amino acid residues, *i*, with other vertices in a protein structure. *C*_*i*_, the clustering coefficient of the amino acid residue, *i*, is defined as the fraction of links that exist among the nearest neighbors of the amino acid residue, *i*, to the maximum number of possible links among them. Therefore,

$$\begin{array}{c}{{\boldsymbol{C}}}_{{\boldsymbol{i}}}=\frac{{\bf{2}}{{\boldsymbol{n}}}_{{\boldsymbol{i}}}}{{{\boldsymbol{k}}}_{{\boldsymbol{i}}}({{\boldsymbol{k}}}_{{\boldsymbol{i}}}-{\bf{1}})}\end{array}$$

where *n*_*i*_ is the number of links that actually exist, and *k*_*i*_ (*k*_*i*_ −1)/2 is the number of all possible links for the nearest neighbors. *C* of a network can be calculated by

$$\begin{array}{c}{\boldsymbol{C}}=\frac{{\bf{1}}}{{\boldsymbol{N}}}\mathop{\sum }\limits_{{\boldsymbol{i}}={\bf{1}}}^{{\boldsymbol{N}}}{{\boldsymbol{C}}}_{{\boldsymbol{i}}}\end{array}$$

*L* is defined as

$$\begin{array}{c}{\boldsymbol{L}}=\frac{{\bf{2}}}{{\boldsymbol{N}}({\boldsymbol{N}}-{\bf{1}})}\mathop{\sum }\limits_{{\boldsymbol{i}}={\bf{1}}}^{{\boldsymbol{N}}-{\bf{1}}}\mathop{\sum }\limits_{{\boldsymbol{j}}={\boldsymbol{i}}+{\bf{1}}}^{{\boldsymbol{N}}}{{\boldsymbol{L}}}_{{\boldsymbol{ij}}}\end{array}$$

where *L*_*ij*_ is the number of links on the shortest path between the *i*th and *j*th amino acid vertices. We also calculated additional parameters in network, assortativity and maximum vertex degree (*k*_max_). Assortativity is the correlation of the degree between vertices adjacent to each other^34^. *k*_max_ is the largest *k* in the network.

### Data sets of protein structures

We obtained protein structures from the PDB database^16^. Our study focused on four broad protein structural classes—all-α, all-β, α + β and α/β, according to the SCOP classification^10^. SCOP^9–11^ is the oldest and manual-based approach and classifies a single αβ class of CATH^12,13^ into two classes, α + β and α/β. To acquire detailed information about relationship between structural classes and network properties, we used the SCOP classification data to examine the difference between α + β and α/β classes by the network parameters. All-α proteins comprise predominantly α-helices, while all-β proteins, β sheets. α + β proteins have α-helices and β-strands that are largely segregated, whereas α/β proteins are largely interspersed^10^. To ensure the quality of structural data used for AAN construction, we selected high-resolution (higher than 2.0 Å) X-ray crystallographic structures of proteins as classified all-α, all-β, α + β and α/β in SCOP. In low resolution protein structures (less than 2.0 Å), considerable ambiguity in the atomic coordinates was found, where chemical groups such as phenyl rings and carboxylates) were not resolved^35^. We also omitted the protein and domain structures including any ligands or nucleic acids, due to the difficulty in definition of such non-amino acid components in AAN. In Table 2, the number of protein and domain structures which satisfied the above criteria are shown. Although the SCOP database has approximately 10,000 protein and domain structures in the all-α, all-β, α + β and α/β classes, the number of the high-resolution protein and domain structures showing higher resolution (2.0 Å) without non-amino acid components is less than 2,000 structures. Furthermore, some protein structures were not converted into the specific AAN structures due to errors in the construction of the AAN. These errors are caused by the lack of atomic coordinates at the middle of the sequence in the PDB entry files. In our study, we removed these randomly occurring irregular data sets.

The difference in the number of structures in data sets mainly arises from the different requirement of the specific atomic coordinate for AANs (Table 2). In construction of the ADN, a link can be established even if the atomic coordinates of some other atoms are lost. On the other hand, construction of the CAN requires all the atomic coordinates of the Cα atoms for each amino acid residue. Therefore, the data set of CAN has the smaller number of structures than that of the ADN. Due to the more complex definition of the ISN, the ISN has a smaller number of structures in the data set than the CAN and the ADN. While the number of structures in data sets for these three methods are not identical, most of structures in data sets are common in these three models and only a few structures are included in one or two of them (Fig. S1). The analysis was, therefore, performed using the common data set in the three models. The results shown in Figs 2–7, using the common data set, are quite similar to the analysis using whole data set for each model shown in Figs S4–S9, where the numbers of the datasets are different among the models.

To construct the learning data set to estimate the classification probability, two selection criteria are added to the criteria for the data listed in Tables 1 and S1. We omitted modified proteins to avoid the effect of non-amino acid components on the AAN structure. The protein structures with less than 90% coverage of a protein sequence^16,36^ were also excluded from the leaning data set. Table S2 exhibits the PDB entries and the numbers of the proteins used for the learning data set. The results shown in Figs S10–S15 are similar to those using the whole data set shown in Figs S4–S9. The analysis we performed here was, therefore, not biased against data set.

## Supplementary information

Supplementary Materials (Figures and Tables)
