# Supplementary material for: Quantitative description and classification of protein structures by a novel robust amino acid network: interaction selective network (ISN)
Source: Sci Rep. 2019 Nov 13;9:16654. doi: 10.1038/s41598-019-52766-6 (PMC6853966; doi:10.1038/s41598-019-52766-6)
Supplement: Supplementary file 1 — Supplementary Materials (Figures and Tables) [file 41598_2019_52766_MOESM1_ESM.docx]

Supplementary Materials

**Quantitative description and classification of protein structures by a novel robust amino acid network: interaction selective network (ISN)**

Shohei Konno^1^, Takao Namiki^2^ & Koichiro Ishimori^1,3^

^1^Graduate School of Chemical Sciences and Engineering, Hokkaido University, Sapporo, 060-8628, Japan. ^2^Department of Mathematics, Faculty of Science, Hokkaido University, Sapporo, 060-0810, Japan. ^3^Department of Chemistry, Faculty of Science, Hokkaido University, Sapporo, 060-0810, Japan. Correspondence and requests for materials should be addressed to K.I. (email: koichiro@sci.hokudai.ac.jp)

**Supplementary Table S1** Lists of PDB IDs of protein structures and structural domains for constructing amino acid networks (AANs). PDB IDs used for the interaction selective network (ISN), the Cα network (CAN) and atom distance network (ADN) are listed. The numbers of the structures are shown in Table 2. All listed structures are included in the *testing* data set of the logistic regression analysis.

| Class | Data set for | | | PDB ID |
| --- | --- | --- | --- | --- |
|  | ISN | CAN | ADN |  |
| All-α | Yes | Yes | Yes | 1A62, 1AA2, 1BEA, 1BKR, 1BZ4, 1CEM, 1DVO, 1EG3, 1EG4, 1ELK, 1ENJ, 1ENK, 1EYH, 1EZ3, 1FAZ, 1FPO, 1FQI, 1GAK, 1GS9, 1GXN, 1GXQ, 1HYP, 1I2T, 1IAP, 1J8M, 1JMW, 1K0M, 1K6K,1KLX, 1KW4, 1LJP, 1LKI, 1LWB, 1MIX, 1MN8, 1MZL, 1N7N, 1N7O, 1N7P, 1NC5, 1NG6, 1NTY, 1OAI, 1OPC, 1OUV, 1OXJ, 1P2F, 1PBV, 1PBW, 1Q5Z, 1R0D, 1R69, 1RJ1, 1S29, 1SBX, 1T6O, 1T95, 1TQG, 1TZV, 1U2K, 1UJ8, 1VAP, 1VIN, 1W7B, 1WER, 1WWI, 1X91, 1XQO, 1XSZ, 1Y6I, 1YU5, 1YU8, 1Z96, 1ZLB, 2CWY, 2CXD, 2D5J, 2END, 2ES9, 2F23, 2FF4, 2FQ3, 2GV2, 2ICT, 2IJQ, 2IOL, 2IXM, 2J5Y, 2J9V, 2LIS, 2OEB, 2P5K, 2Q0Z, 2QJZ, 2QY9, 2RH3, 2RJV, 2RJY, 2VE8, 2YGS, 1A8O, 1AIE, 1AIL, 1BGF, 1BHD, 1BM9, 1C02, 1C26, 1CEI, 1CUN, 1D9C, 1DJ8, 1FC3, 1FIP, 1FP3, 1G2Y, 1G2Z, 1G39, 1G8E, 1G8Q, 1GTO, 1GU9, 1HF8, 1IJY, 1IRQ, 1IZM, 1JB6, 1K8U, 1KSO, 1LDD, 1LJ9, 1M4R, 1NKD, 1NOG, 1NP3, 1O0W, 1OTK, 1Q2H, 1QQF, 1QSJ, 1R1T, 1R1U, 1R7J, 1RK4, 1ROP, 1S4K, 1SGM, 1SH5, 1T06, 1UFB, 1UFI, 1UNK, 1UTG, 1UZ3, 1VLS, 1WU9, 1X2I, 1XSV, 1XWG, 1Y6X, 1Y7Y, 1YIB, 1YIG, 1ZKR, 1ZTD, 2A61, 2A8F, 2B0J, 2CWL, 2D8E, 2DB7, 2F6L, 2FD5, 2FQ4, 2G7O, 2G7S, 2G9E, 2GEN, 2GMY, 2GPO, 2GS4, 2GZ4, 2IB0, 2IJK, 2O4D, 2O70, 2OFY, 2OKU, 2OO2, 2PMR, 2SPC, 2UTG, 2ZA7, 3C90, 3LYN, 3WRP |
|  | No | Yes | Yes | 1H13, 1H14, 1KS9, 1KU3, 1L8R, 1LIS, 1ORC, 1SH6, 1SQG, 1U00, 1VKU, 1WLZ, 1XW2, 1XWT, 1YZM, 2FEZ, 2HUJ, 2IOS, 1AYI, 1B6Q, 1BAZ, 1CI4, 1DKX, 1DKZ, 1GMG, 1ILK, 1MD0, 1RFY, 1RPO, 1TJV, 1V2Z, 1XG7, 1YNB, 2A6B, 2FBN, 2FBQ, 2GOM, 2HZT, 2O8P, 2PEQ, 2Q0T |
|  | No | No | Yes | 1E6I, 1EA8, 1H7I, 1FBQ, 1FBS, 1FBU, 1QJA, 1VKA |
|  | Yes | No | Yes | 1GVJ, 1UPG, 1US6, 1UTU, 2IU5, 2ZBS |
|  | No | Yes | No | 1NIG, 2CJJ, 1C3C, 1H99, 1O3U |
|  | Yes | Yes | No | 2CKX, 1BGF |
|  | Yes | No | No | --- |

| Class | Data set for | | | PDB ID |
| --- | --- | --- | --- | --- |
|  | ISN | CAN | ADN |  |
| All-β | Yes | Yes | Yes | 1A58, 1A62, 1AAJ, 1AGJ, 1AME, 1AMM, 1AMX, 1ARB, 1AUN, 1AW7, 1AWQ, 1AWR, 1B7I, 1B7J, 1BCK, 1BFG, 1BHE, 1BJ7, 1BK1, 1C5H, 1CDY, 1CKA, 1CKB, 1CQY, 1CRZ, 1CWC, 1CWF, 1CWH, 1CWI, 1CWJ, 1CWK, 1CWL, 1CWM, 1CWO, 1CYN, 1CZT, 1DDJ, 1DDV, 1DDW, 1DSL, 1DUA, 1DUE, 1DYW, 1E5P, 1EDQ, 1EG3, 1EG4, 1EKL, 1ENX, 1EQV, 1EUJ, 1EUR, 1EVH, 1EY0, 1EY4, 1EY5, 1EY6, 1EY8, 1EY9, 1EYA, 1EYC, 1EYD, 1EZ6, 1EZ8, 1F00, 1F2M, 1F2Y, 1F2Z, 1FC6, 1FC7, 1FC9, 1FGL, 1FKN, 1FL0, 1FNF, 1FSO, 1G1K, 1GCS, 1GMU, 1GSM, 1GZI, 1H6T, 1HOE, 1HV0, 1HV1, 1I0C, 1IDK, 1IFB, 1IFC, 1IFG, 1IHZ, 1II3, 1IOB, 1IST, 1IU1, 1IV8, 1IZ6, 1J2A, 1J48, 1JAB, 1JB3, 1JPE, 1KAA, 1KAB, 1KEX, 1KHI, 1KMT, 1KN3, 1KQX, 1KYF, 1KYU, 1L8F, 1LB6, 1LMI, 1LOP, 1LPJ, 1LPL, 1M1G, 1M1S, 1MBM, 1MD6, 1MFG, 1MFL, 1MHN, 1MIK, 1MIX, 1MJC, 1N7E, 1N7N, 1N7O, 1N7P, 1NG2, 1NG5, 1NKR, 1NOA, 1NTY, 1OA4, 1OGM, 1OLR, 1OPS, 1P3C, 1P4P, 1PDQ, 1PP3, 1PSO, 1PVX, 1PWT, 1PXZ, 1PZC, 1Q3L, 1Q7F, 1QCX, 1QKX, 1QOI, 1QRZ, 1QTP, 1QTS, 1QWX, 1QZN, 1R8N, 1RI6, 1ROW, 1RWR, 1SGZ, 1SHG, 1SLL, 1SNO, 1SQW, 1SSH, 1STN, 1SYC, 1SYE, 1SYG, 1T2P, 1THV, 1TS3, 1TUD, 1TVQ, 1TWB, 1UAI, 1UCS, 1UEB, 1UNP, 1UOK, 1UTI, 1UXZ, 1VAV, 1VBS, 1VCA, 1VDN, 1W8V, 1WGB, 1WKA, 1WOS, 1WP5, 1WWC, 1X6L, 1XN2, 1XN3, 1XNC, 1XND, 1XO7, 1XYO, 1XYP, 1YHF, 1YNA, 1YRZ, 1YTQ, 1Z0H, 1Z78, 1ZCE, 21BI, 2A6Z, 2AME, 2BEN, 2BIT, 2BJQ, 2BVV, 2BXX, 2CNY, 2CNZ, 2CO4, 2CPL, 2CV3, 2EIF, 2ERF, 2ES3, 2F15, 2FCB, 2FF4, 2FU0, 2H3L, 2I1B, 2I6V, 2J43, 2JIA, 2JIC, 2MSI, 2MSJ, 2NUZ, 2OEI, 2PCY, 2PIE, 2R99, 2RA2, 2SFA, 2SIL, 2SPG, 2YXF, 31BI, 3APP, 3APR, 3BDU, 3DJ9, 3EZM, 3MSI, 3TSS, 4GCR, 4I1B, 4MSI, 4PEP, 5MSI, 6MSI, 7AME, 7MSI, 8AME, 9AME, 1A12, 1A1X, 1AB0, 1AE2, 1AE3, 1ALY, 1AT0, 1B0W, 1B2P, 1B8E, 1BFT, 1BKB, 1BKZ, 1BMZ, 1BRE, 1BWW, 1BZ8, 1BZD, 1BZE, 1C1F, 1C3K, 1C48, 1CDC, 1CQ3, 1D2O, 1DQ0, 1DUN, 1DUP, 1DVQ, 1E15, 1E65, 1EP0, 1EZG, 1F39, 1F41, 1FCG, 1FH2, 1FIV, 1G6G, 1GD7, 1GKH, 1GPR, 1GVP, 1H64, 1H6U, 1HIX, 1HQQ, 1HXL, 1HXZ, 1HY2, 1HZ9, 1HZA, 1I07, 1I81, 1I8D, 1IS5, 1IX2, 1JHC, 1JVK, 1K32, 1KFF, 1KHX, 1KL3, 1KL4, 1KL5, 1KNB, 1KU8, 1KZQ, 1L7J, 1LGV, 1LIB, 1LVE, 1MGQ, 1N7F, 1NLR, 1NQB, 1NXM, 1NXZ, 1O6A, 1OBQ, 1OBU, 1OGH, 1OKI, 1OPA, 1OV3, 1PM4, 1Q5U, 1QAC, 1QB5, 1QWD, 1REI, 1RST, 1RSU, 1SFP, 1SHF, 1SLE, 1SLG, 1SMX, 1SOK, 1STM, 1STR, 1STS, 1SVP, 1SWA, 1SWB, 1SWL, 1SWO, 1TCZ, 1TD4, 1TH7, 1TSH, 1TTA, 1TTB, 1TTC, 1TTR, 1TVD, 1U1S, 1U1T, 1UDZ, 1UJ1, 1UZ2, 1V1H, 1V6Z, 1V8H, 1VIE, 1VPN, 1VSC, 1VWA, 1VWB, 1VWC, 1VWD, 1VWE, 1VWF, 1VWG, 1VWH, 1VWM, 1VWN, 1VWO, 1VWP, 1W9A, 1WHI, 1WHO, 1WLG, 1WLT, 1WTL, 1XB9, 1XDH, 1XHN, 1XXO, 1Y2T, 1YFN, 1Z8K, 2A13, 2AS0, 2CGA, 2DLB, 2DP9, 2DUC, 2ED6, 2FHQ, 2G4G, 2GEC, 2GQV, 2HPE, 2J2J, 2J71, 2MCG, 2NPH, 2OUJ, 2OX7, 2OYZ, 2P84, 2PSG, 2PYT, 2Q4I, 2Q4N, 2QGB, 2RHE, 2TRH, 2TRY, 3D3R, 3MCG |

| Class | Data set for | | | PDB ID |
| --- | --- | --- | --- | --- |
|  | ISN | CAN | ADN |  |
| All-β  (Continued) | No | Yes | Yes | 1ACX, 1B9K, 1BAS, 1BE9, 1EY7, 1FNA, 1G9O, 1GYU, 1GYV, 1HCV, 1I04, 1I1B, 1I1J, 1IHJ, 1KDC, 1L6P, 1L7R, 1L6P, 1L7R, 1MSI, 1NPU, 1PE9, 1PEV, 1PGS, 1PHT, 1QAU, 1RFE, 1TEN, 1TJ6, 1TP3, 1TP5, 1TQ3, 1TS9, 1TSF, 1U00, 1U6D, 1UH9, 1V9T, 1VAI, 1VLO, 1VPR, 1WBA, 1XVM, 1ZGK, 1ZMF, 2BEN, 2BTL, 2BYG, 2FEZ, 2FNE, 2FR2, 2G30, 2I1B, 2RMC, 3SEB, 1A30, 1AOH, 1CJD, 1CZY, 1D00, 1D01, 1D0A, 1DKX, 1DKZ, 1E3F, 1EEQ, 1EI5, 1FHN, 1FON, 1FUX, 1IGQ, 1LCL, 1MR7, 1MY5, 1MY7, 1N99, 1OHQ, 1PQE, 1SEM, 1TD0, 1TY0, 1U3Z, 1VQA, 1VQB, 1VQC, 1VQD, 1VQE, 1VQF, 1VQG, 1VQH, 1VQI, 1VQJ, 1XE0, 1YIF, 2D0N, 2F9H, 2GIY, 2PAB, 2Q8O, 3CBR, 3D7P, 4TSV |
|  | No | No | Yes | 1HBQ, 1KDA, 1KDB, 1OGM, 2BHG, 1UPI, 2P23 |
|  | Yes | No | Yes | 2VO8 |
|  | No | Yes | No | 1FHG, 1G4M, 1MJS, 1RZ2, 1A64 |
|  | Yes | Yes | No | 1MS3, 1MS5, 2Q0Z, 2OUJ |
|  | Yes | No | No | 1SND |

| Class | Data set for | | | PDB ID |
| --- | --- | --- | --- | --- |
|  | ISN | CAN | ADN |  |
| α+β | Yes | Yes | Yes | 132L, 133L, 134L, 135L, 153L, 180L, 1ACF, 1AGI, 1AHC, 1AKI, 1AKO, 1AW7, 1B1J, 1B6V, 1BB3, 1BD8, 1BHF, 1BK7, 1BM8, 1BMB, 1BOL, 1BVX, 1BWH, 1BWI, 1BWJ, 1C44, 1CEW, 1CKH, 1CM2, 1CM3, 1CNS, 1CQM, 1CS8, 1D4T, 1D4W, 1DIX, 1DKJ, 1DZF, 1EDQ, 1EIC, 1EID, 1EIE, 1EKG, 1EM7, 1EQ6, 1EW4, 1F00, 1F0W, 1F10, 1FLY, 1FS3, 1FU0, 1FVA, 1G61, 1GBS, 1GCU, 1GMU, 1GND, 1GOU, 1GX3, 1HEL, 1HEM, 1HEN, 1HEO, 1HEP, 1HEQ, 1HER, 1HKA, 1HSW, 1HSX, 1HUF, 1HZT, 1I1Z, 1I20, 1I7K, 1IGD, 1IOQ, 1IOR, 1IOS, 1IOT, IQ4, 1IR7, 1IR8, 1IR9, 1IV7, 1IV9, 1IXL, 1IZP, 1IZQ, 1IZR, 1J27, 1J74, 1JHS, 1JIS, 1JIT, 1JIY, 1JJ1, 1JJ3, 1JOS, 1JVW, 1JWR, 1JYH, 1JYR, 1K1A, 1K1B, 1K50, 1KAF, 1KF5, 1KF7, 1KF8, 1KHP, 1KHQ, 1KOE, 1KXW, 1KXX, 1KXY, 1KYF, 1KYU, 1L01, 1L02, 1L04, 1L05, 1L06, 1L07, 1L08, 1L09, 1L10, 1L12, 1L13, 1L14, 1L15, 1L16, 1L17, 1L18, 1L19, 1L20, 1L21, 1L22, 1L23, 1L24, 1L25, 1L27, 1L28, 1L29, 1L30, 1L31, 1L32, 1L33, 1L34, 1L35, 1L37, 1L38, 1L39, 1L40, 1L41, 1L42, 1L43, 1L44, 1L45, 1L46, 1L47, 1L49, 1L50, 1L51, 1L52, 1L54, 1L60, 1L97, 1LAA, 1LHH, 1LHI, 1LHJ, 1LHK, 1LHL, 1LHM, 1LIT, 1LKL, 1LL7, 1LLN, 1LMN, 1LN4, 1LOU, 1LOZ, 1LP8, 1LSA, 1LSB, 1LSC, 1LSD, 1LSE, 1LSF, 1LSM, 1LSN, 1LSY, 1LTU, 1LYD, 1LYO, 1LYS, 1LYY, 1LYZ, 1LZ1, 1LZA, 1LZD, 1LZT, 1M1G, 1MLA, 1MOL, 1MWP, 1MX4, 1MX6, 1NIJ, 1NWA, 1O0X, 1OGW, 1OQW, 1OSD, 1OZ9, 1P1L, 1P56, 1P7S, 1PGB, 1PIP, 1PQK, 1PRZ, 1PV5, 1PXW, 1Q2Y, 1QS1, 1QS9, 1QSW, 1QTH, 1QTP, 1QTS, 1QXT, 1QY3, 1QYO, 1R4B, 1R9H, 1RA4, 1RAT, 1RBX, 1RC9, 1REX, 1RFP, 1RHA, 1RL0, 1RL6, 1ROA, 1SC0, 1SHA, 1SHB, 1SMB, 1SQH, 1SQW, 1T07, 1T2I, 1T3X, 1T95, 1TAY, 1TBY, 1TCY, 1TDY, 1TF1, 1TIG, 1TJE, 1TP6, 1TS3, 1TUA, 1U79, 1U9A, 1U9B, 1UBI, 1UBQ, 1UCO, 1UEK, 1UIC, 1UID, 1UIE, 1UIF, 1UIG, 1ULR, 1UOH, 1VAJ, 1VCC, 1VDQ, 1VDS, 1VDT, 1VED, 1VF8, 1VFQ, 1WDV, 1WNA, 1WOS, 1X6L, 1XIX, 1XIZ, 1XKR, 1XPS, 1XPT, 1XSZ, 1Y0O, 1Y6L, 1YCK, 1YH2, 1YI6, 1YPC, 1YQB, 1ZPW, 1ZWZ, 1ZZK, 214L, 256L, 2A4D, 2AIF, 2AUB, 2BAA, 2BF5, 2BQG, 2BQH, 2BQI, 2BQK, 2BQM, 2C8O, 2C8P, 2CDS, 2CW4, 2CZW, 2D4O, 2D4P, 2DYJ, 2ESK, 2ESO, 2ESQ, 2F23, 2FAZ, 2FC3, 2FZP, 2G8Q, 2GS5, 2HRX, 2IGD, 2LHM, 2LYM, 2LYZ, 2LZM, 2NR7, 2NWD, 2O0P, 2OHW, 2P4X, 2POF, 2PPN, 2PV1, 2RAT, 2RER, 2YVB, 2YVT, 2ZD2, 2ZEQ, 2ZJD, 2ZQ4, 3BZP, 3BZR, 3BZT, 3CTK, 3LYM, 3LYZ, 3LZM, 3RAT, 3RSD, 3TSS, 4LYT, 4LYZ, 4RAT, 5LYT, 5LYZ, 5RAT, 6LYT, 6LYZ, 6RAT, 7RAT, 8RAT, 9RAT, 137L, 1A3A, 1A68, 1B8P, 1BHH, 1BUO, 1BV1, 1BV4, 1BXY, 1BYR, 1CGQ, 1DHN, 1DPT, 1DSX, 1E15, 1EL6, 1EO6, 1ERZ, 1ESR, 1EYM, 1F2L, 1F46, 1F9Q, 1F9R, 1FTR, 1GFL, 1GIF, 1GXJ, 1GY6, 1GYB, 1HFO, 1HLW, 1HQ8, 1I7N, 1IDP, 1IFT, 1IHB, 1IV1, 1IZ9, 1J2V, 1J8B, 1KCM, 1KPA, 1KPB, 1KPT, 1KR4, 1KS2, 1M4J, 1M5S, 1MBY, 1MMI, 1MP9, 1MSC, 1N5B, 1NAP, 1NFJ, 1NPK, 1O0W, 1O22, 1O5J, 1OCV, 1OK7, 1OTF, 1PCF, |

| Class | Data set for | | | PDB ID |
| --- | --- | --- | --- | --- |
|  | ISN | CAN | ADN |  |
| α+β  (Continued) | Yes | Yes | Yes | 1Q6H, 1Q8R, 1QAH, 1QDV, 1QTO, 1QU9, 1QVE, 1QW2, 1QWI, 1R29, 1R7L, 1RWZ, 1RXZ, 1SEI, 1SH8, 1SPH, 1T1D, 1T4A, 1T4D, 1T82, 1TE5, 1TFE, 1TKI, 1TLU, 1TMI, 1TVX, 1TWU, 1TY0, 1U07, 1U69, 1U7I, 1VDH, 1VFJ, 1VGG, 1VH5, 1VLA, 1VPK, 1VQ3, 1W5R, 1WKJ, 1WM3, 1WZ3, 1X25, 1XS0, 1Y5H, 1Y9W, 1YAL, 1YER, 1YLX, 1YQH, 1YVO, 1Z4E, 1ZO2, 2A10, 2B3M, 2B5R, 2CB5, 2CHC, 2CHS, 2CU6, 2CVL, 2CWK, 2D3G, 2D7V, 2E2C, 2F5G, 2FB5, 2FIU, 2FL4, 2FUJ, 2G3T, 2GDG, 2GDQ, 2GE7, 2GR8, 2GUK, 2HIQ, 2HNG, 2IKB, 2IPR, 2IVY, 2J6B, 2J7Z, 2JER, 2NCK, 2NML, 2NMU, 2NWV, 2O5U, 2O7M, 2OMO, 2PII, 2PV2, 2RFR, 2RK5, 3D1E, 3IL8 |
|  | No | Yes | Yes | 1AFU, 1B9K, 1BNF, 1BOX, 1BRI, 1BSA, 1BSB, 1BSC, 1BSE, 1DEU, 1DTJ, 1DZO, 1EOE, 1F32, 1FLQ, 1FLU, 1FLW, 1FN5, 1FUS, 1FZY, 1G24, 1GQZ, 1GZ2, 1HHL, 1HZ6, 1ICX, 1IS0, 1JBB, 1KH0, 1LCJ, 1LZ4, 1MHX, 1MI0, 1OJQ, 1PGX, 1PNE, 1PTF, 1RHB, 1T3Y, 1UKF, 1UN3, 1VDP, 1VHF, 1VHS, 1VLO, 1X6P, 1X6Q, 1X6R, 1X6X, 1X6Y, 1X6Z, 1XTE, 1YHW, 1YPA, 1YPB, 1ZHV, 2F1N, 2FHT, 2FO3, 2G30, 2O0Q, 2PST, 3BU3, 3BU6, 3LYZ, 3SEB, 1A09, 1A1A, 1AH6, 1BKP, 1FNJ, 1FNK, 1GY7, 1JB2, 1JD1, 1JYA, 1JYQ, 1JZO, 1KWB, 1MFF, 1MK4, 1MKB, 1MZG, 1Q8B, 1R0V, 1RG0, 1RIS, 1RU0, 1RW0, 1S7I, 1TKL, 1U0K, 1VAX, 1VGY, 1VKN, 1WM2, 2AAG, 2AAL, 2C2I, 2CI2, 2G3A |
|  | No | No | Yes | 1E21, 1M1H, 1UMW, 1UYL, 1VJK, 1VR9, 1W41, 1W42, 1W9G, 2BSZ, 2Q43, 1H8X, 1I4J, 2DM9 |
|  | Yes | No | Yes | 1H9O, 1W3E, 2V94, 1O7Z, 1O80, 1ODE |
|  | No | Yes | No | 1K4N, 2CXA |
|  | Yes | Yes | No | 1P4O |
|  | Yes | No | No | 1QYU |

| Class | Data set for | | | PDB ID |
| --- | --- | --- | --- | --- |
|  | ISN | CAN | ADN |  |
| α/β | Yes | Yes | Yes | 1A8Q, 1AK1, 1AKZ, 1ARL, 1ATZ, 1B00, 1B31, 1BN6, 1BQC, 1C4W, 1CEX, 1CHD, 1CNV, 1CRZ, 1CUA, 1CUB, 1CUC, 1CUF, 1CUG, 1CUH, 1CUJ, 1CUS, 1CUU, 1CUX, 1CUY, 1CWY, 1CZ1, 1DIN, 1DUS, 1DZF, 1E0W, 1E6L, 1E6M, 1EDE, 1EDG, 1EDQ, 1EQP, 1EUG, 1EZK, 1F21, 1F9M, 1FAA, 1FBN, 1FC6, 1FC7, 1FC9, 1FG4, 1G8A, 1GCU, 1GND, 1GOA, 1GOB, 1GOC, 1GZJ, 1H1N, 1H4Y, 1H6T, 1H75, 1I1X, 1I39, 1I60, 1IIB, 1IUK, 1IUL, 1IV8, 1J8M, 1JFR, 1JFU, 1JL1, 1JLN, 1JR2, 1JXB, 1JYK, 1K0M, 1K6A, 1KVA, 1KVB, 1KVC, 1LAV, 1LAW, 1LL7, 1LU4, 1LZL, 1M21, 1MF7, 1MLA, 1MPB, 1N3Y, 1NA5, 1NAR, 1NIJ, 1NM8, 1NY1, 1O13, 1O7U, 1O85, 1O8W, 1O9G, 1ONR, 1OTM, 1P15, 1P1X, 1P2F, 1PGV, 1PRY, 1Q0U, 1Q2U, 1Q7S, 1QK8, 1QWK, 1QZ0, 1QZM, 1R88, 1RBR, 1RBS, 1RBT, 1RBU, 1RBV, 1RDB, 1RH9, 1RHS, 1SRV, 1SU9, 1T1U, 1T7N, 1THX, 1TIB, 1TM2, 1TMY, 1TR9, 1TUX, 1U24, 1U9C, 1UC7, 1UOK, 1UXO, 1V77, 1VF8, 1W94, 1WD7, 1WDE, 1WEH, 1WOU, 1WSJ, 1X42, 1X6L, 1XFK, 1XYZ, 1XZA, 1XZE, 1XZF, 1XZG, 1XZH, 1XZI, 1XZJ, 1YXY, 1YZF, 1ZON, 2A4V, 2AH5, 2APJ, 2B3S, 2BV9, 2CHF, 2CVB, 2CXH, 2CYG, 2D59, 2EXO, 2F9F, 2F9S, 2FG1, 2FHP, 2FI9, 2HAD, 2HVM, 2IIY, 2NX2, 2ORA, 2PKY, 2PLC, 2PTD, 2PTH, 2QXT, 2QXU, 2QY9, 2R48, 2RN2, 2YWO, 2YXP, 3E9O, 3ENB, 3TGL, 4EUG, 1A4U, 1A7U, 1A88, 1ADE, 1AIU, 1AJR, 1ALD, 1AUO, 1B43, 1B8P, 1BI5, 1BSL, 1BYI, 1DQZ, 1DSB, 1DTS, 1DYS, 1E15, 1E5M, 1EDT, 1ERT, 1ERV, 1ERW, 1ES9, 1F5Z, 1FSF, 1G2Q, 1GQN, 1H6U, 1H7E, 1HM5, 1HQK, 1HRD, 1HXH, 1I45, 1I7N, 1I89, 1I8B, 1ILV, 1IU8, 1IZ9, 1J22, 1J23, 1J2W, 1J33, 1J7G, 1J85, 1JFL, 1JIQ, 1JKM, 1JWX, 1JZO, 1K32, 1KS2, 1KTN, 1KZ1, 1L7A, 1LU9, 1LX7, 1M6J, 1N7K, 1NP3, 1NS5, 1NSW, 1NTH, 1NXV, 1NXZ, 1O63, 1OCK, 1OQF, 1P5F, 1PBN, 1PDO, 1PDV, 1Q5X, 1Q98, 1QMV, 1QVZ, 1R0S, 1R12, 1S2T, 1SUR, 1T4D, 1T5O, 1T6T, 1TC5, 1TCD, 1TFU, 1TVN, 1U0V, 1UCF, 1UDR, 1UFO, 1UIU, 1V5X, 1V6Z, 1V7L, 1V8E, 1VC1, 1VGY, 1VHC, 1VHV, 1WDJ, 1XK6, 1XK7, 1XMP, 1XVW, 1XWG, 1XXU, 1YAC, 1YE5, 1ZAH, 2AB0, 2AS0, 2B0A, 2B0J, 2B61, 2CAR, 2DST, 2DVP, 2FUK, 2GDQ, 2GTD, 2I5D, 2I5I, 2I9I, 2O57, 2OB5, 2R1U, 2VB7, 2VL3, 3BED, 3CT6, 3ERJ, 3PGA, 6XIA |
|  | No | Yes | Yes | 1A2J, 1A3H, 1AGY, 1C3P, 1CV2, 1EWX, 1FFA, 1FFB, 1FFC, 1FFD, 1FFE, 1FTO, 1G1F, 1IJB, 1IO2, 1IXK, 1JW4, 1KID, 1KNG, 1KS9, 1L7R, 1M5T, 1MTZ, 1NDB, 1O20, 1O6D, 1OMP, 1QO2, 1R26, 1SQG, 1T15, 1T1J, 1UAX, 1VI4, 1VIC, 1VL5, 1YTL, 2EXO, 2ISB, 2LAO, 1AC1, 1ACV, 1AC1, 1ACV, 1BED, 1ECP, 1EPX, 1F2J, 1FVK, 1IHC, 1JL2, 1O4W, 1PE0, 1PRX, 1R3R, 1RK4, 1SOA, 1VGA, 1VHX, 1VKN, 1VR6, 1WOH, 1YPI, 2AHB, 2F7W, 2FA8, 2GUB, 2JK2 |
|  | No | No | Yes | 1LL7, 1W94, 1Y9U, 1FBA, 1USG, |
|  | Yes | No | Yes | 1GOK, 1GOM, 1O8X, 1OEM, 1GP1, 1O9N, 1O9Q, 1OBJ, 1OCH, 1OJX, 1QCZ, 1QKK, 2BGK |
|  | No | Yes | No | 1J7X, 1R0V, |
|  | Yes | Yes | No | --- |
|  | Yes | No | No | 1ZCU, |

**Supplementary Table S2** Lists of PDB IDs of protein structures and structural domains for constructing amino acid networks (AANs) in the *learning* data set of the logistic regression analysis. PDB IDs used for the interaction selective network (ISN), the Cα network (CAN) and atom distance network (ADN) are listed. The numbers of structures are shown in Table 2.

| Class | Data set for | | | PDB ID |
| --- | --- | --- | --- | --- |
|  | ISN | CAN | ADN |  |
| All-α | Yes | Yes | Yes | 1uj8, 1hyp, 1klx, 2end, 1tzv, 1ng6, 2oeb, 1ljp, 1y6i, 1vap, 1xqo, 1ouv, 2f23, 2ijq, 1nc5, 1xsz, 2d5j, 1nkd, 1rpo, 2oo2, 2pmr, 1cei, 1r7j, 3wrp, 1y7y, 2za7, 2fd5, 2a8f, 1kso, 2fbq, 1bm9, 1ztd, 1lj9, 1c02, 1unk, 2b0j, 3c90, 1sgm, 1r1u, 1t06, 1otk, 1o0w, 1ufb, 2a61, 2cwl, 1b43, 1fp3, 2gz4, 2gmy, 2o70, 1np3 |
|  | No | Yes | Yes | 1ks9, 2fez, 1h13, 1sqg, 1ayi, 1cuk, 1xg7, 1ynb |
|  | No | No | Yes | --- |
|  | Yes | No | Yes | 1us6 |
|  | No | Yes | No | --- |
|  | Yes | Yes | No | --- |
|  | Yes | No | No | --- |

| Class | Data set for | | | PDB ID |
| --- | --- | --- | --- | --- |
|  | ISN | CAN | ADN |  |
| All-β | Yes | Yes | Yes | 1ucs, 1ame, 1mjc, 1tvq, 1ifb, 1ifc, 1lpj, 1kqx, 2eif, 1md6, 1w8v, 2cpl, 1awq, 1dyw, 1amm, 1gcs, 4gcr, 1qoi, 1kn3, 1r8n, 1sqw, 1xnd, 2jic, 2sfa, 1pvx, 1row, 2cv3, 3app, 1ist, 1qcx, 1wos, 1ueb, 1bhe, 1iz6, 1pp3, 1vav, 1uok, 1pxz, 1m1g, 1awr, 1yrz, 1gvp, 1a1x, 1whi, 2dp9, 1lib, 1is5, 2p84, 1c3k, 1gpr, 1ep0, 1v8h, 1opa, 1bkz, 1y2t, 1xxo, 1wlt, 1nxm, 1gd7, 1cq3, 2cga, 1i81, 1mgq, 1ku8, 1i8d, 1e15, 1th7, 1a12, 1h64, 1yif, 1k32 |
|  | No | Yes | Yes | 2fr2, 1hk0, 1wba, 1xvm, 2fez, 1pev, 1pe9, 1vqb, 1lcl, 1ei5, 1cjd, 1mr7 |
|  | No | No | Yes | 1snt |
|  | Yes | No | Yes | --- |
|  | No | Yes | No | --- |
|  | Yes | Yes | No | --- |
|  | Yes | No | No | --- |

| Class | Data set for | | | PDB ID |
| --- | --- | --- | --- | --- |
|  | ISN | CAN | ADN |  |
| α+β | Yes | Yes | Yes | 1ulr, 1zpw, 1j27, 1p1l, 1ln4, 1ew4, 2ppn, 1ra4, 2czw, 2fc3, 1acf, 1tp6, 1dkj, 1q2y, 1t3x, 2d4p, 1vfq, 1j74, 2esk, 1oz9, 1bd8, 1jyh, 1hka, 1u9a, 1u9b, 2a4d, 1lyd, 2lzm, 3lzm, 1rl6, 153l, 1gbs, 1mol, 1sqw, 2dyj, 1tua, 1iv9, 1cqm, 1nwa, 1xkr, 1dzf, 1rc9, 1bol, 1uoh, 2baa, 1zwz, 1pxw, 1o0x, 2ohw, 1ako, 1uek, 2zjd, 1oqw, 1gcu, 1ltu, 1mla, 2f23, 1nij, 1xiz, 1xix, 1wos, 1vf8, 1gnd, 1g61, 2p4x, 1xsz, 1ll7, 1m1g, 1gx3, 1nfj, 1ris, 2pii, 1bxy, 1dhn, 1qto, 1s7i, 1twu, 2fl4, 1npk, 1bv1, 2b3m, 1r7l, 2hiq, 1eo6, 1reg, 1rwz, 1gy6, 1rxz, 1x25, 1zo2, 1sei, 2f5g, 1kcm, 1qah, 1wkj, 1y9w, 2nck, 1vh5, 1z4e, 1sh8, 1mk4, 2cwk, 1ihb, 1b8p, 1mkb, 2gdg, 1cgq, 1gif, 1dpt, 1otf, 1mp9, 1jzo, 1gfl, 1rw0, 1o0w, 1te5, 1n5b, 1erz, 2fe5, 1tkl, 1iz9, 1el6, 1hfo, 1mmi, 3d1e, 1ok7, 2gdq, 1iv1, 1e15, 1ftr, 1vdh |
|  | No | Yes | Yes | 1ptf, 1box, 2o0q, 1hhl, 1zhv, 1t3y, 1icx, 1ojq, 2f1n, 1gqz, 1jbb, 1et6, 2nml, 1q8r, 1tlu, 2g3t, 1ode, 1vq3, 1vpk, 2o5u, 1bkp, 1ty0, 2a10, 1jd1 |
|  | No | No | Yes | 1vjk, 1w9g |
|  | Yes | No | Yes | --- |
|  | No | Yes | No | --- |
|  | Yes | Yes | No | --- |
|  | Yes | No | No | --- |

| Class | Data set for | | | PDB ID |
| --- | --- | --- | --- | --- |
|  | ISN | CAN | ADN |  |
| α/β | Yes | Yes | Yes | 1h75, 1thx, 1tmy, 1wou, 1e6l, 2chf, 2fi9, 1iuk, 1iul, 2d59, 1srv, 1o7u, 1o85, 1o8w, 1o8x, 1ezk, 2rn2, 1goa, 2nx2, 1q2u, 2pth, 1yzf, 1v77, 1i39, 1g8a, 1eug, 1h4y, 1tib, 1a8q, 1gcu, 1bqc, 1k6a, 1mla, 1ak1, 1ede, 2had, 2pky, 2yxp, 2cyg, 1tm2, 1qwk, 1nij, 1lzl, 1xfk, 1mpb, 1omp, 1rh9, 1vf8, 1gnd, 1yxy, 1cwy, 1p1x, 1wd7, 1uok, 1jr2, 1gzj, 1onr, 1t1u, 1ll7, 1ert, 1j7g, 1tfu, 1j85, 2b0a, 2dvp, 1p5f, 1vc1, 2fuk, 1byi, 1v8e, 3erj, 1gqn, 2dst, 3ct6, 1fsf, 1ye5, 1v7l, 1b8p, 1q98, 1j33, 2b0j, 1ald, 1g2q, 2r1u, 1dsb, 6xia, 2gub, 2car, 1v5x, 2ab0, 1iu8, 1e5m, 1jzo, 1auo, 1jfl, 1n7k, 1h7e, 1ypi, 1tcd, 1ktn, 1a4u, 1udr, 1a7u, 1wdj, 1oqf, 1s2t, 1xxu, 1l7a, 1bsl, 1iz9, 1b43, 1dys, 2ahb, 1jkm, 2gdq, 1hkq, 1ock, 1a88, 1lu9, 1ade, 1j2w, 1e15, 1hxh, 1hm5, 1hrd, 1np3, 1xmp, 1t5o, 1zah, 2gtd, 1woh, 1jiq, 1k32 |
|  | No | Yes | Yes | 1m5t, 1r26, 1o6d, 1a2j, 1io2, 1t1j, 1ks9, 1mtz, 1cv2, 1jw4, 1sqg, 1uax, 1vic, 1ytl, 2ex0, 2i5d, 1f2j, 1fvk, 1ucf, 1q5x, 1mjf, 1tc5, 1aug, 1vr6, 1ecp, 1epx, 1vl2 |
|  | No | No | Yes | 1qk8 |
|  | Yes | No | Yes | 2bgk, 1ojx |
|  | No | Yes | No | --- |
|  | Yes | Yes | No | 1l2l |
|  | Yes | No | No | --- |

**Supplementary Figure S1** Distribution of network parameters of interaction selective network (ISN) (A, B), α-carbon network (CAN) (C, D) and atom distance network (AND) (E, F). The distribution was calculated by using *common* data set of the three network models. Average distance(*L*)-average clustering coefficient (*C*) plots (A, C, E) and maximum vertex degree (*k*_max_)-vertex assortativity plots (B, D, F) are shown.

**Supplementary Figure S2** The plots of the average clustering coefficient (*C*) and the average vertex degree (*k*) of the interaction selective network (ISN) with different cutoff value (*R*_c_) of the hydrogen bonds. The distribution was calculated by using *individual whole* data set in the ISN (1556 structures). *R*_c_ is set as (A) 2.0 Å, (B) 2.2 Å, (C) 2.4 Å, (D) 2.6 Å, (E) 2.8 Å, (F) 3.0 Å, (G) 3.2 Å, (H) 3.4 Å, (I) 3.5 Å, (J) 3.6 Å, (K) 3.8 Å, (L) 4.0 Å, (M) 4.2 Å, (N) 4.4Å, (O) 4.6 Å, (P) 4.8 Å, and (Q) 5.0 Å. Plots of all-α and all-β proteins are colored in red and blue, respectively.

**Supplementary Figure S3** The plots of the average clustering coefficient (*C*) and the average vertex degree (*k*) of the interaction selective network (ISN) with different cutoff value (*R*_c_) of the hydrophobic interaction. The distribution was calculated by using *individual whole* data set in the ISN (1556 structures). *R*_c_ is set as (A) 3.5 Å, (B) 3.6 Å, (C) 3.8 Å, (D) 4.0 Å, (E) 4.2 Å, (F) 4.4 Å, (G) 4.6 Å, (H) 4.8 Å, (I) 5.0 Å, (J) 5.2 Å, (K) 5.4 Å, (L) 5.6 Å, (M) 5.8 Å, (N) 6.0Å, (O) 6.2 Å, (P) 6.4 Å, and (Q) 6.5 Å. Plots of all-α and all-β proteins are colored in red and blue, respectively.

**Supplementary Figure S4** The plots of the average clustering coefficient (*C*) and the average vertex degree (*k*) in an of the interaction selective network (ISN) with different cutoff value (*R*_c_) of the hydrogen bond. The distribution was calculated by using *individual whole* data set in the ISN (1556 structures). The *R*_c_ is set as (A) 3.2 Å, (B) 3.4 Å, (C) 3.8 Å, and (D) 5.0 Å. Plots of all-α and all-β proteins are colored in red and blue, respectively. In (C), discriminant line, $C=0.915-0.0564 k$ was determined by the logistic regression analysis of the common data set of the three network models.


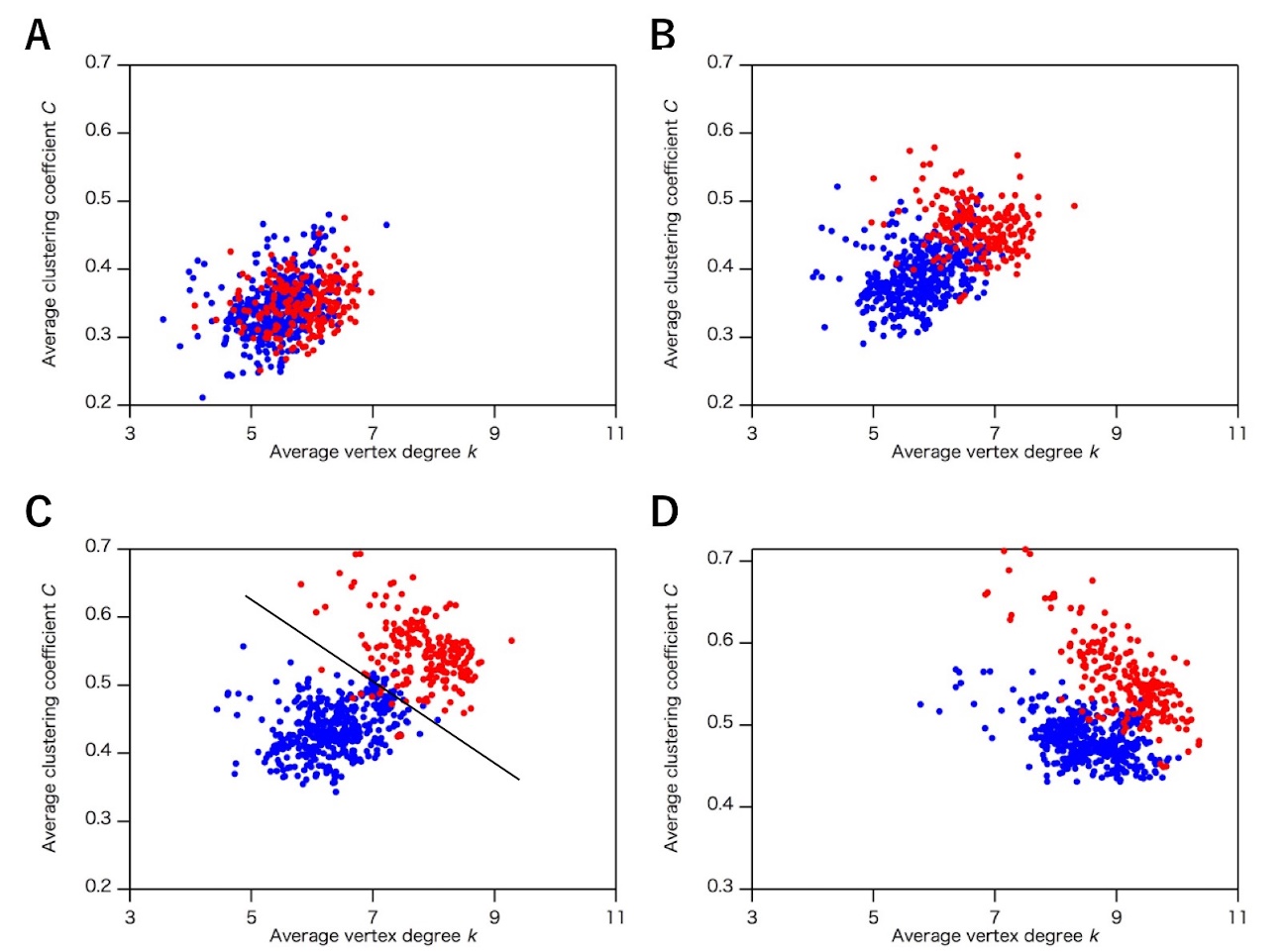


**Supplementary Figure S5** The plots of the average clustering coefficient (*C*) and the average vertex degree (*k*) of the interaction selective network (ISN) with different cutoff value (*R*_c_) of the hydrophobic interaction. The distribution was calculated by using *individual whole* data set in the ISN (1556 structures). The *R*_c_ is set as (A) 4.0 Å, (B) 5.0 Å, and (C) 6.0 Å. Plots of all-α and all-β proteins are colored in red and blue, respectively.


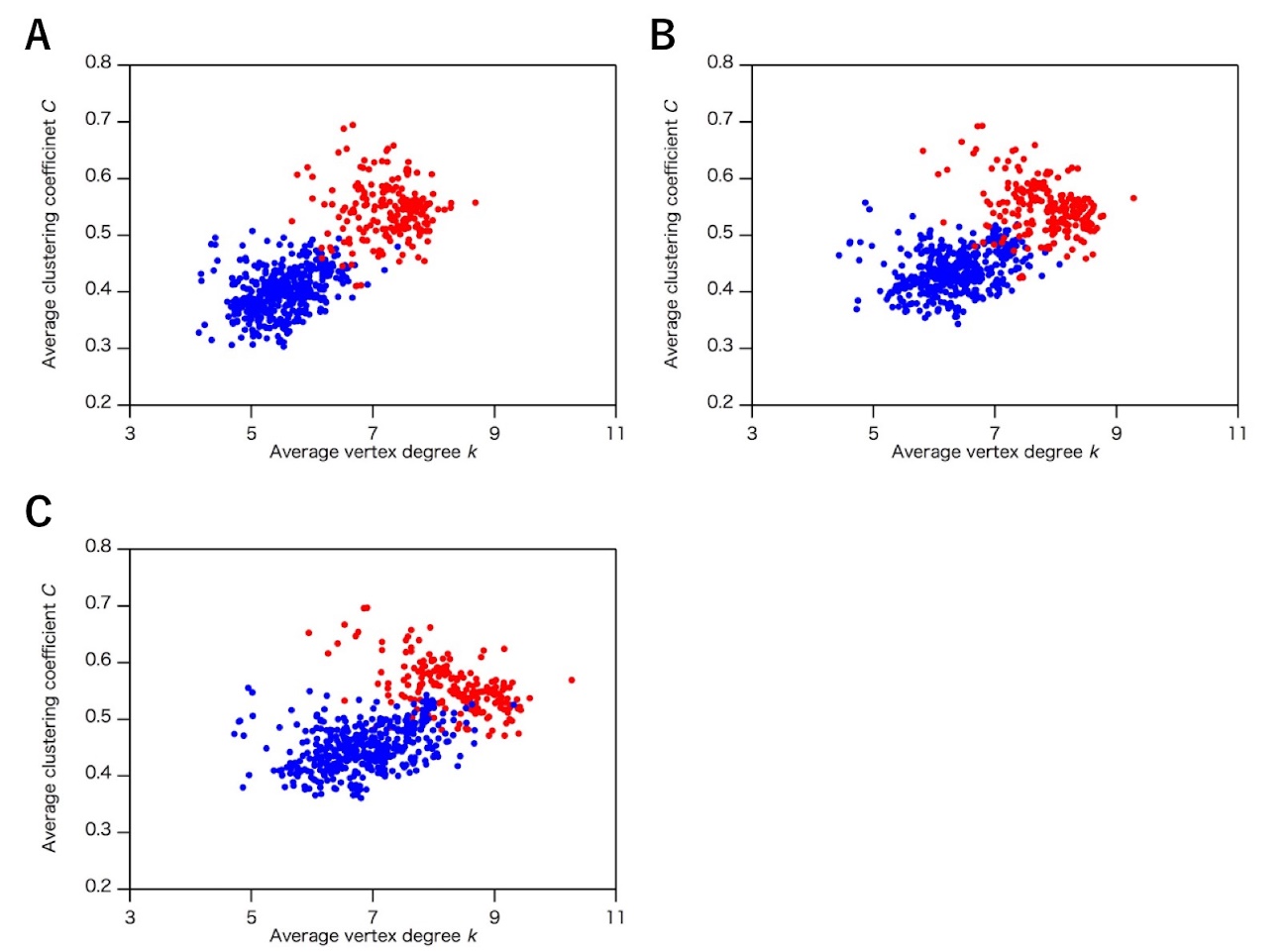


**Supplementary Figure S6** (A) The plot of average clustering coefficient (*C*) and average vertex degree (*k*) of the interaction selective network (ISN). The distribution was calculated by using *individual whole* data set in the ISN (1556 structures). Protein structures are categorized as all-α (red), all-β (blue), α+β (green), and α/β (orange); (B)(C) *k*-*C* plots identical to (A) with visualizing (B) α-helix and (C) β-sheet content. Secondary structure content is shown with colored plot as follows; 0% (pink), 1%–10% (red), 11%–20% (orange), 21%–30% (yellow green), 31%–40% (green), 41%–50% (light blue), 51%–60% (blue), 61%–70% (purple), 71%–80% (deep purple), 81%–90% (grey), and 91%–99% (black). In (B) and (C), these protein structures are classified as all-α ($\circ$), all-β ($\times$), α+β ($+$), and α/β ($\triangle$).


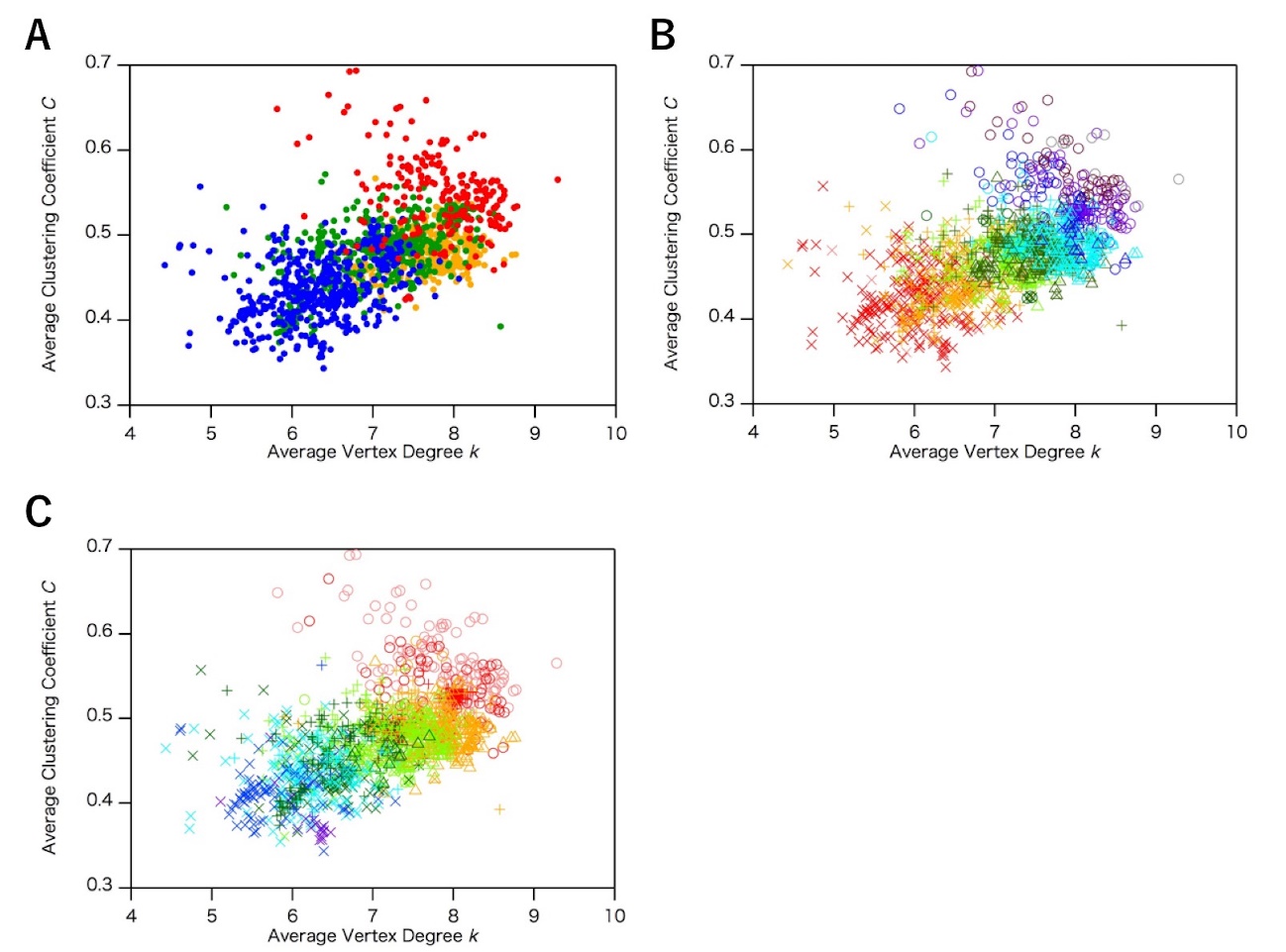


**Supplementary Figure S7** The plot of average clustering coefficient (*C*) and average vertex degree (*k*) of (A) CAN (*R*_c_ = 8.5 Å) and (B) ADN. The distribution was calculated by using *individual whole* data sets in the CAN (1850 structures) and the ADN (1890 structures). The protein class of each protein is shown with colored plot as follows: all-α (red), all-β (blue), α+β (green), and α/β (orange).


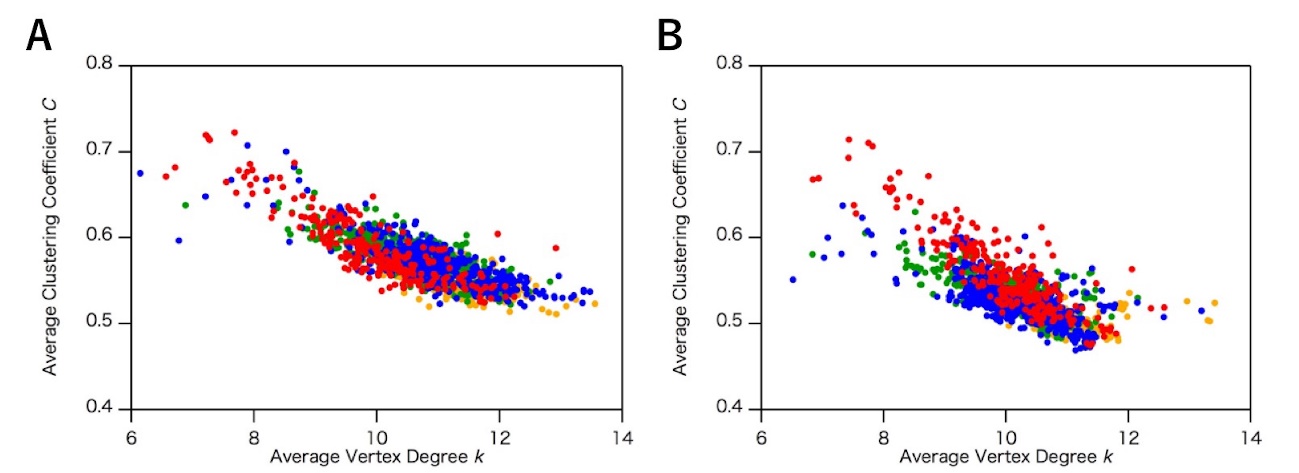


**Supplementary Figure S8** The plot of the number of links (*N*_Links_) and the number of vertices (*N*_Vertices_) of (A) ISN, (B) CAN (*R*_c_ = 8.5 Å), and (C) ADN (*R*_c_ = 5.0 Å). The distribution was calculated by using the *individual whole* data sets. The class of each protein is shown with colored plot as follows: all-α (red), all-β (blue), α+β (green), and α/β (orange).


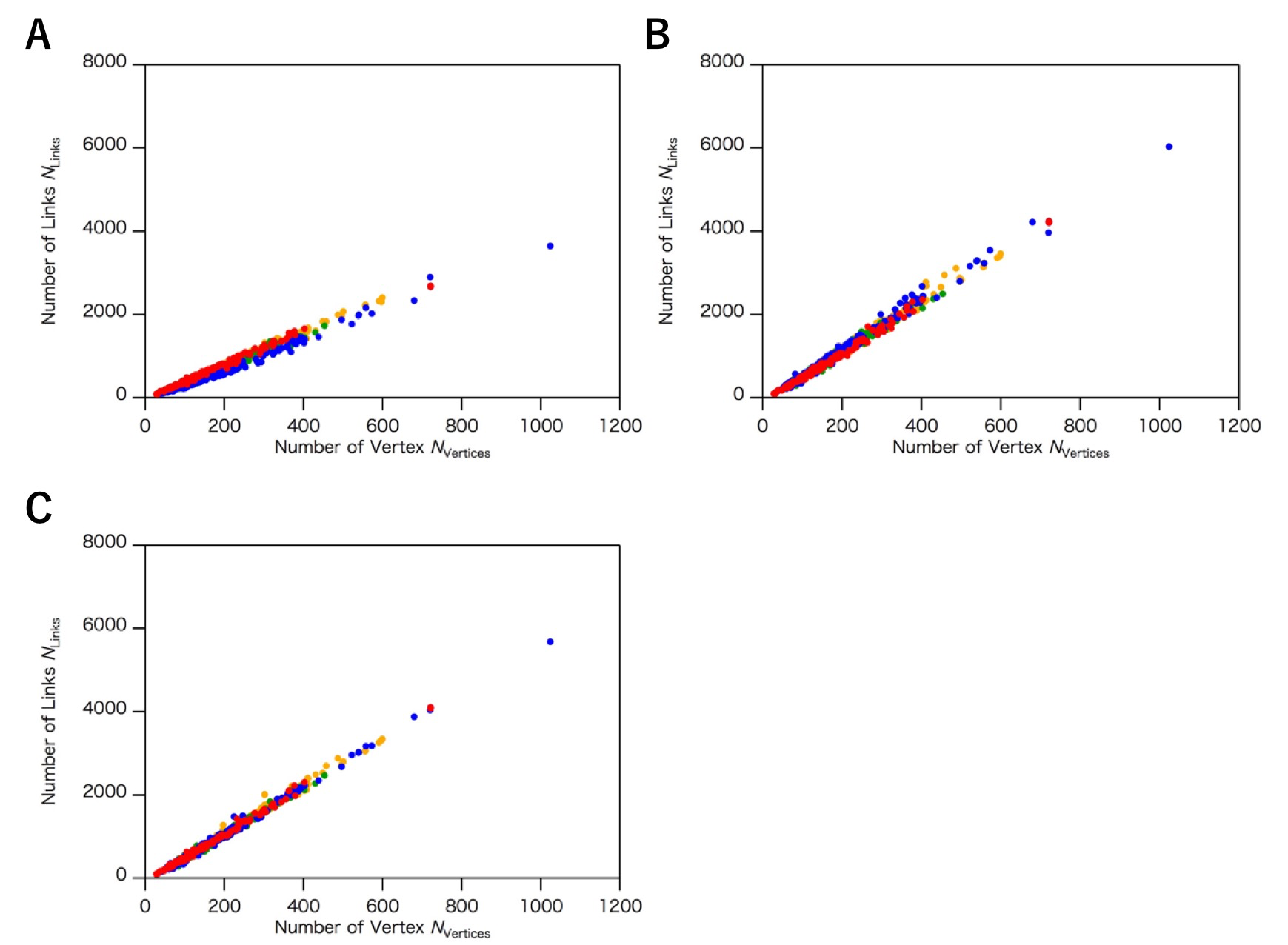


**Supplementary Figure S9** Average clustering coefficient (*C*)–average vertex degree (*k*) plots (A)–(D) and average distance *L*–*C* plots (E) of CAN. The distribution was calculated by using *individual whole* data set in the CAN (1850 structures). The cutoff value (*R*_c_) of CAN is (A), (E) 7.0 Å, (B) 6.0 Å, (C) 5.5 Å, and (D) 5.0 Å. All-α protein structures are represented as red circles and all-β protein structures are shown as blue circles. In (C), discriminant line, $C=0.619-0.0593 k$ was determined by the logistic regression analysis of the common data set of the three network models.


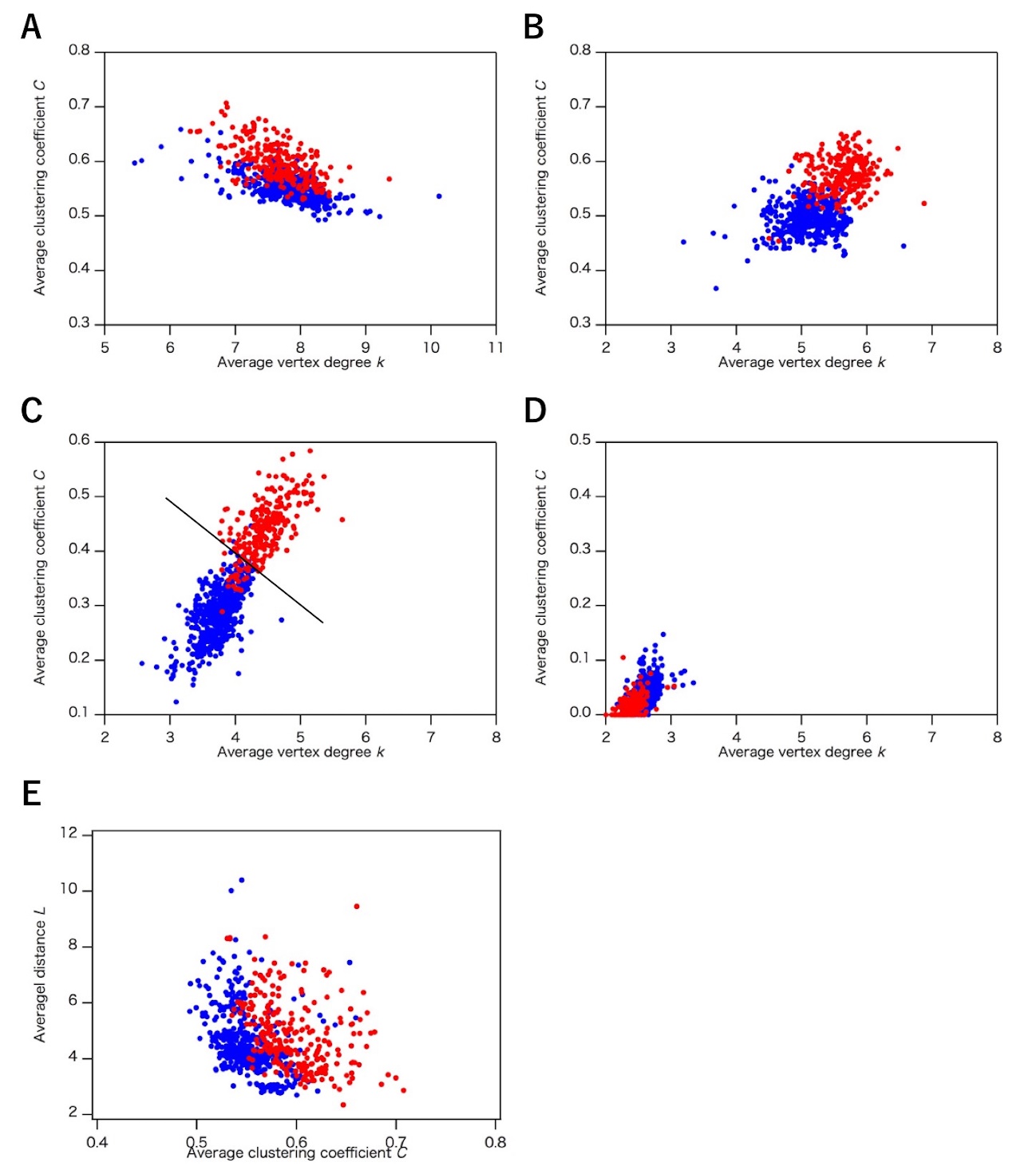


**Supplementary Figure S10** The plots of the average clustering coefficient (*C*) and the average vertex degree (*k*) in an of the interaction selective network (ISN) with different cutoff value (*R*_c_) of the hydrogen bond. The distribution was calculated by using the *learning* data set. The *R*_c_ is set as (A) 3.2 Å, (B) 3.4 Å, (C) 3.8 Å, and (D) 5.0 Å. Plots of all-α and all-β proteins are colored in red and blue, respectively.

**Supplementary Figure 11** The plots of the average clustering coefficient (*C*) and the average vertex degree (*k*) of the interaction selective network (ISN) with different cutoff value (*R*_c_) of the hydrophobic interaction. The distribution was calculated by using the *learning* data set. The *R*_c_ is set as (A) 4.0 Å, (B) 5.0 Å, and (C) 6.0 Å. Plots of all-α and all-β proteins are colored in red and blue, respectively.

**Supplementary Figure S12** (A) The plot of average clustering coefficient (*C*) and average vertex degree (*k*) of the interaction selective network (ISN) . The distribution was calculated by using the *learning* data set. Protein structures are categorized as all-α (red), all-β (blue), α+β (green), and α/β (orange); (B)(C) *k*-*C* plots identical to (A) with visualizing (B) α-helix and (C) β-sheet content. Secondary structure content is shown with colored plot as follows; 0% (pink), 1%–10% (red), 11%–20% (orange), 21%–30% (yellow green), 31%–40% (green), 41%–50% (light blue), 51%–60% (blue), 61%–70% (purple), 71%–80% (deep purple), 81%–90% (grey), and 91%–99% (black). In (B) and (C), these protein structures are classified as all-α ($\circ$), all-β ($\times$), α+β ($+$), and α/β ($\triangle$).

**Supplementary Figure S13** The plot of average clustering coefficient (*C*) and average vertex degree (*k*) of (A) CAN (*R*_c_ = 8.5 Å) and (B) ADN. The distribution was calculated by the *learning* data sets. The protein class of each protein is shown with colored plot as follows: all-α (red), all-β (blue), α+β (green), and α/β (orange).

**Supplementary Figure S14** The plot of the number of links (*N*_Links_) and the number of vertices (*N*_Vertices_) of (A) ISN, (B) CAN (*R*_c_ = 8.5 Å), and (C) ADN (*R*_c_ = 5.0 Å). The distribution was calculated by using the *learning* data sets. The class of each protein is shown with colored plot as follows: all-α (red), all-β (blue), α+β (green), and α/β (orange).

**Supplementary Figure S15** Average clustering coefficient (*C*)–average vertex degree (*k*) plots (A)–(D) and average distance *L*–*C* plots (E) of CAN. The distribution was calculated by using the *learning* data sets. The cutoff value (*R*_c_) of CAN is (A), (E) 7.0 Å, (B) 6.0 Å, (C) 5.5 Å, and (D) 5.0 Å. All-α protein structures are represented as red circles and all-β protein structures are shown as blue circles.
